# ^89^Zr-immuno-PET using the anti-LAG-3 tracer [^89^Zr]Zr-BI 754111: demonstrating target specific binding in NSCLC and HNSCC

**DOI:** 10.1007/s00259-023-06164-w

**Published:** 2023-03-02

**Authors:** Iris H.C. Miedema, Marc C. Huisman, Gerben J.C. Zwezerijnen, Rolf Grempler, Alejandro Perez Pitarch, Andrea Thiele, Raphael Hesse, Mabrouk Elgadi, Alexander Peltzer, Danielle J. Vugts, Guus A.M.S. van Dongen, Tanja D. de Gruijl, C. Willemien Menke-van der Houven van Oordt, Idris Bahce

**Affiliations:** 1 Department of Medical Oncology, Amsterdam UMC location Vrije Universiteit Amsterdam, De Boelelaan 1117, 1081 HV Amsterdam, the Netherlands; 2 Imaging and Biomarkers, Cancer Center Amsterdam, De Boelelaan 1117, 1081 HV Amsterdam, the Netherlands; 3 Department of Radiology and Nuclear Medicine, Amsterdam UMC location Vrije Universiteit Amsterdam, De Boelelaan 1117, 1081 HV Amsterdam, the Netherlands; 4 Department of Translational Medicine & Clinical Pharmacology, Boehringer Ingelheim Pharmaceuticals, 900 Ridgebury Road, Ridgefield, CT 06877 USA; 5 Department of Translational Medicine & Clinical Pharmacology, Boehringer Ingelheim Pharma GmbH & Co. KG, Birkendorfer Strasse 65, 88400 Biberach and der Riss, Germany; 6 Cancer Biology and Immunology, Cancer Center Amsterdam, De Boelelaan 1117, 1018 HV Amsterdam, the Netherlands; 7 Department of Pulmonary Medicine, Amsterdam UMC location Vrije Universiteit Amsterdam, De Boelelaan 1117, 1081 HV Amsterdam, the Netherlands

**Keywords:** LAG-3, PD-1, PET, Zirconium-89, Immunotherapy, TILs

## Abstract

**Purpose:**

Although lymphocyte activation gene-3 (LAG-3) directed therapies demonstrate promising clinical anti-cancer activity, only a subset of patients seems to benefit and predictive biomarkers are lacking. Here, we explored the potential use of the anti-LAG-3 antibody tracer [^89^Zr]Zr-BI 754111 as a predictive imaging biomarker and investigated its target specific uptake as well as the correlation of its tumor uptake and the tumor immune infiltration.

**Methods:**

Patients with head and neck (*N* = 2) or lung cancer (*N* = 4) were included in an imaging substudy of a phase 1 trial with BI 754091 (anti-PD-1) and BI 754111 (anti-LAG-3). After baseline tumor biopsy and [^18^F]FDG-PET, patients were given 240 mg of BI 754091, followed 8 days later by administration of [^89^Zr]Zr-BI 754111 (37 MBq, 4 mg). PET scans were performed 2 h, 96 h, and 144 h post-injection. To investigate target specificity, a second tracer administration was given two weeks later, this time with pre-administration of 40 (*N* = 3) or 600 mg (*N* = 3) unlabeled BI 754111, followed by PET scans at 96 h and 144 h post-injection. Tumor immune cell infiltration was assessed by immunohistochemistry and RNA sequencing.

**Results:**

Tracer uptake in tumors was clearly visible at the 4-mg mass dose (tumor-to-plasma ratio 1.63 [IQR 0.37-2.89]) and could be saturated by increasing mass doses (44 mg: 0.67 [IQR 0.50–0.85]; 604 mg: 0.56 [IQR 0.42–0.75]), demonstrating target specificity. Tumor uptake correlated to immune cell-derived RNA signatures.

**Conclusions:**

[^89^Zr]Zr-BI-754111 PET imaging shows favorable technical and biological characteristics for developing a potential predictive imaging biomarker for LAG-3-directed therapies.

**Trial registration:**

ClinicalTrials.gov, NCT03780725. Registered 19 December 2018

**Supplementary Information:**

The online version contains supplementary material available at 10.1007/s00259-023-06164-w.

## Introduction

Lymphocyte activation gene 3 (LAG-3, CD233) is an inhibitory receptor that can be found on the surface of T-cells, Tregs, NK-cells, plasmacytoid dendritic cells, and tumor-associated macrophages [1–3]. The binding of LAG-3 to its canonical ligand, major histocompatibility protein II, initiates inhibitory downstream signaling in T-cells and can contribute to immune escape [4, 5]. Expression of LAG-3 can be found on cells in the tumor microenvironment (TME) of various solid tumors, including non-small cell lung cancer (NSCLC) and head and neck squamous cell carcinomas (HNSCC), and is commonly associated with a more aggressive tumor type and poor prognosis [6, 7]. Moreover, upregulation of LAG-3 expression on tumor-infiltrating lymphocytes (TILs) has been suggested as a resistance marker to anti-programmed death 1 (anti-PD-1) treatment in patients who initially respond well to this treatment [8, 9]. In light of these findings, LAG-3 has been studied extensively as a potential druggable target—especially for dual checkpoint blockade with anti-PD-1 and anti-LAG-3—which has yielded promising results in preclinical studies [10–13]. Even more encouraging, a recent clinical study demonstrated that relatlimab (an anti-LAG-3 monoclonal antibody [mAb]) in combination with nivolumab (an anti-PD-1 mAb) improved progression-free survival in first-line advanced melanoma patients over nivolumab monotherapy, consequently resulting in Food and Drug Administration approval of this treatment [14]. Currently, over 100 clinical trials comprising LAG-3 blocking treatments are ongoing in various solid and hematological malignancies [9].

Although anti-LAG-3 is a promising immune checkpoint inhibitor (ICI), not all patients benefit and there is a clear clinical need for the development of predictive biomarkers. One modality that could provide such biomarkers is positron emission tomography (PET) using radiolabeled mAbs, which we introduced previously using the positron emitter ^89^Zr, i.e., ^89^Zr-immuno-PET [15]. The potential of ^89^Zr-immuno-PET to evaluate the biodistribution and tumor uptake of ICIs is being demonstrated in an ever-growing number of clinical trials. Two separate studies involving radiolabeled anti-PD-1 mAbs ([^89^Zr]Zr-nivolumab [16] and [^89^Zr]Zr-pembrolizumab [17], showed that a higher tumor uptake correlated with a favorable treatment outcome. Similarly, tumor uptake with [^89^Zr]Zr-atezolizumab (anti-programmed death ligand 1 [anti-PD-L1] mAb) positively correlated with response to atezolizumab treatment, outperforming immunohistochemistry (IHC) in this regard [18]. Although these were pilot studies with limited sample sizes, they highlight the potential of ^89^Zr-immuno-PET to generate predictive biomarkers for ICIs.

To apply the ^89^Zr-immuno-PET method to an anti-LAG-3 mAb would be highly interesting. However, based on the literature, the availability of LAG-3 targets in the tumor microenvironment is expected to be rather low (0.41 ng/mg in cervical tumors) as compared to other targets, such as human epidermal growth factor receptor-2-2 (12–800 ng/mg in breast cancer) or MET (22–700 ng/mg in NSCLC) [19, 20]. This has questioned whether an anti-LAG-3 PET tracer would yield a sufficiently quantifiable and target-specific signal for LAG-3 expression. Despite this uncertainty, investigating the biomarker value of LAG-3 PET can be justified, as ^89^Zr-immuno-PET may still perform well due to the residualizing characteristics of ^89^Zr after target-specific binding and internalization of ^89^Zr-mAb, which might result in an enhancement of the PET signal upon synthesis and membrane translocation of new LAG-3 receptors during imaging [21, 22].

Therefore, in this study, we investigated ^89^Zr-immuno-PET imaging using a ^89^Zr-labeled anti-LAG-3 mAb ([^89^Zr]Zr-BI 754111). Firstly, we evaluated the feasibility of visualizing and quantifying the biodistribution and tumor uptake of [^89^Zr]Zr-BI 754111. Secondly, the effect of administering additional unlabeled BI 754111 pre-tracer injection was explored to provide evidence for target-specific uptake [23], and potentially target saturation—which might also help to inform decisions on a recommended phase II dose (RP2D) in the absence of acute toxicities [24]. Thirdly, we correlated tracer uptake to TME immune features through IHC and RNA-sequencing signatures.

## Patients and methods

### Patient selection

Patients with advanced stage HNSCC and NSCLC, who had progressed on previous anti-PD-(L)1-based treatment, after having initially experienced at least 3 months of stable disease on this treatment, were included in this study. Additionally, eligible patients needed to have at least one lesion that was approachable for biopsy and that was evaluable with PET (≥ 20 mm, outside of the liver). Other key inclusion criteria included measurable disease according to Response Evaluation Criteria in Solid Tumours (RECIST) 1.1 and iRECIST, Eastern Cooperative Oncology Group (ECOG) performance status of 0 or 1, life expectancy of at least 12 weeks, and age ≥ 18 years. Exclusion criteria were inadequate organ function or bone marrow reserve, significant cardiovascular disease, history of pneumonitis within the last 5 years, history of interstitial lung disease, history of severe hypersensitivity to other mAbs, history of auto-immune disease excluding vitiligo or resolved childhood asthma/atopy, active infection requiring systemic treatment, history of human immunodeficiency virus infection, active hepatitis B or C virus infection, not having fully recovered from major surgery, necessary major surgery planned 12 months after inclusion, use of restricted medications (defined as any other anticancer agent, systemic immunosuppressive medications including > 10 mg/day of prednisone or equivalent, and/or any herbal medications), planning to receive live attenuated vaccines during the trial, chronic alcohol or drug abuse, and pregnancy or expecting pregnancy during the trial.

### Study design

Screening procedures included a tumor biopsy and a standard 2-deoxy-2-[fluorine-18]fluoro-D-glucose ([^18^F]FDG) PET/computed tomography (CT) scan, performed according to European Association of Nuclear Medicine guidelines 2.0 [25]. Within two weeks after the [^18^F]FDG PET/CT scan, patients received their first treatment cycle, which consisted of 240 mg of the anti-PD-1 antibody ezabenlimab (C1D1), recreating the conditions under which progression initially occurred. One week thereafter (C1D8), the zirconium-89-labelled LAG-3 tracer ([^89^Zr]Zr-BI 754111) was administered, which contained 37 MBq of [^89^Zr]Zr-BI 754111 and 4 mg of BI 754111. Whole-body PET/CT scans were obtained < 2, 90 ± 1, and 138 ± 1 h post-injection (p.i.). Three weeks after the start of the first cycle, patients received the second treatment cycle consisting of 240 mg of ezabenlimab and either 40 mg (*N* = 3) or 600 mg (*N* = 3) of the anti-LAG-3 antibody BI 754111 (C2D1). Directly thereafter (< 2 h), patients received a second administration of [^89^Zr]Zr-BI 754111. PET/CT scans were acquired after 90 ± 1 and 138 ± 1 h. Summarizing, three mass doses of [^89^Zr]Zr-BI 75411 were investigated within this protocol: 4 mg in cycle 1, followed by 44 mg in cycle 2 (*N* = 3) and 4 mg in cycle 1, followed by 604 mg in cycle 2 (*N* = 3). After completion of imaging procedures, patients continued with three-weekly treatment cycles of 240 mg of ezabenlimab and 600 mg of BI 754111 till disease progression or unacceptable toxicity occurred. Baseline CT scans for response assessment were performed either in combination with [^18^F]FDG-PET or 1 week prior to C2D1 and thereafter repeated every 6 weeks. Tumor response was assessed according to RECIST v1.1 and iRECIST criteria [26, 27].

This prospective clinical trial was conducted at Amsterdam UMC, location VUmc, Amsterdam, the Netherlands. All patients provided written informed consent. This study was approved by the Medical Ethics Review Committee of the Amsterdam University Medical Centers and was conducted in accordance with the Declaration of Helsinki, International Conference on Harmonization guidelines, and Good Clinical Practice quality standards [28, 29]. This trial was registered on www.clinicaltrials.gov under the identifier NCT03780725.

### Radioactivity measurements in plasma

Venous blood samples were drawn 1 h p.i. of [^89^Zr]Zr-BI 754111, and after every PET scan. Radioactivity concentration in blood and plasma was measured in a cross-calibrated well counter and expressed as activity concentration (AC) [30].

### Choice of used PET outcome measure

When comparing the effect of different BI 754111 mAb mass doses on [^89^Zr]Zr-BI 754111 tumor uptake, standardized uptake value (SUV) is not considered to be a suitable outcome measure, as it assumes identical clearance between compared groups as well as a distribution volume that is larger than the one known for antibodies [31]. Thus, a relatively low expression as described for LAG-3 as well as small variations in target expression can be overshadowed by temporal differences in plasma availability of the tracer. One way to address this would be to perform a Patlak analysis on tumor data, using the plasma area under the curve as the input function. However, in contrast to normal organs [32], baseline net rate of irreversible uptake (*K*_*i*_) values for tumors lacking target expression are not available so far, i.e., a Patlak analysis cannot be employed to derive *K*_*i*_ values for tumor lesion uptake. Thus, we reported the organ- and tumor-to-plasma ratios, which take the plasma concentration into account. The use and interpretation of tumor-to-plasma ratios continue to be further explored as well as more comprehensive methods to assess antibody tumor uptake. For organs, *K*_*i*_ values are reported.

### Tracer synthesis and quality control

^89^Zr was purchased from Perkin-Elmer, Boston, MA, USA, and coupled to BI 754111 via the bifunctional chelator DFO-Bz-NCS (Macrocyclics) [33]. [^89^Zr]Zr-BI 754111 was produced in compliance with current Good Manufacturing Practice at the Amsterdam UMC, location VUmc. The procedures for radiolabeling of BI 754111 with ^89^Zr have been validated with respect to the final quality of the prepared conjugate and the production process. Details can be found in the supplementary information.

### Scan acquisition

Following low-dose CT scans for attenuation correction, whole-body PET scans were acquired from head to mid-thigh with a scan duration of 5 min per bed position, resulting in a total scan duration of approximately 60 min. PET/CT scans were performed either on a Gemini TOF-64 PET/CT scanner or a Vereos digital PET/CT (both from Philips Medical Systems, Best, The Netherlands).

### Biodistribution analysis and Patlak linearization

To assess biodistribution, organs of interest were delineated, either manually (lungs, spleen, liver, kidneys, bone marrow) or automatically (bone) using the in-house developed BIODISTRIBUTION tool (developed in IDL version 8.4) and reported as organ-to-plasma ratios. In addition, organ activity concentrations in Bq/mL, in combination with activity concentrations from plasma, were used as input for Patlak linearization. The use of the Patlak linearization allows an estimate of reversible and irreversible contributions to measured activity in the region of interest. This approach was previously cross-validated for healthy organs using physiologically based pharmacokinetic models of mAbs [32]. From the Patlak linearization, *K*_*i*_ (in mL·g^−1^·min^−1^), which is a combination of target-specific and non-specific irreversible uptake, was extracted and reported.

### Analysis of tumor uptake of [^89^Zr]Zr-BI 754111

To analyze tumor uptake of [^89^Zr]Zr-BI 754111, we used the in-house developed software ACCURATE [34] and followed a previously described standardized manual procedure for tumor segmentation [35]. In short, tumor lesions were scored by an experienced nuclear medicine physician (GZ) who was initially blinded for [^18^F]FDG-PET and diagnostic CT images. Tumor lesions on the [^89^Zr]Zr-BI 754111 PET images were considered visually positive when focal uptake exceeded the local background. For quantification, max. 5 tumor lesions per patient were selected, with a diameter of at least 2 cm to minimize partial volume effects [36]. Volumes of interest were delineated on the PET image by the physician-researcher (IM) and verified by the nuclear medicine physician (GZ). For tumor accumulation, we report tumor-to-plasma ratios based on peak activity concentration.

### LAG-3 IHC

IHC procedures and scoring for LAG-3 staining were carried out by Roche Diagnostics (Tucson, AZ, USA) using BenchMark ULTRA instrumentation and clone 17B4 (Abcam). Hematoxylin and eosin (H&E) slides were evaluated for tumor content and tissue quality. A minimum of 100 viable tumor cells was required for evaluation. LAG-3 staining was assessed for evaluability based on tissue and cell viability, morphology, and the presence of discernable background staining and scored as the percentage of positive immune cells per tumor area (0–100%). The tumor area included both intra-tumoral and peri-tumoral stroma.

### RNA purification from FFPE

RNA was extracted by using the RNeasy® formalin-fixed, paraffin-embedded (FFPE) Kit (Qiagen, Hilden, Germany) following the manufacturer’s instructions. The starting material for RNA extraction was 10 FFPE sections with a 4–5-μm thickness each. First, paraffin was removed from FFPE tissue sections by treating with 1 mL of xylene. Next, samples were incubated at 56 °C in 150 μL of lysis buffer (+ 10 μL of proteinase K), to release RNA from the sections. A short incubation at 80 °C partially reversed formalin crosslinking of the released nucleic acids, improving RNA yield and quality, as well as RNA performance in downstream RNASeq library preparation. This was followed by DNase treatment (10 μL of 1500 units DNase) to eliminate genomic DNA, including very small DNA fragments. Thereafter, the lysate was mixed with 320 μL of a guanidine salt-containing buffer. A total of 720 μL of ethanol absolute was added to provide appropriate binding conditions for RNA, and the sample was then pipetted onto a RNeasy MinElute spin column (Qiagen, Hilden, Germany), where the total RNA bound to the membrane and contaminants were washed out. RNA was then eluted in 20 μL of RNase-free water.

Determination of RNA concentration and quality check was performed by absorbance measurements at 260 nm and 280 nm using a NanoDrop ND-1000 spectrophotometer (Thermo Fisher Scientific, Waltham, MA, USA). The OD260/OD280 ratio was between 1.7 and 2.3.

### RNA sequencing library preparation and sequencing

Sequencing libraries were prepared from 100 ng of total RNA by using the TruSeq Stranded Total RNA Library Prep Kit with Ribo-Zero Gold according to the manufacturer’s instructions (Illumina Inc., San Diego, CA, USA). Briefly, ribosomal RNA (rRNA) was removed using 5 μL biotinylated, target-specific oligos combined with 35 μL Ribo-Zero rRNA removal beads. A total of 8.5 μL of Elute-Prime-Fragment High Mix was added. As further fragmentation of FFPE RNA is not necessary, it was continued with first-strand synthesis. The RNA fragments were copied into first-strand cDNA using 8 μL of a mixture of 1 μL of SuperScript II reverse transcriptase and 9 μL of First Strand Synthesis Act D Mix (containing random primers), followed by second-strand cDNA synthesis using 20 μL of Second Strand Marking Master Mix containing DNA Polymerase I and RNase H. A single “A” nucleotide is added to the 3′ ends of the blunt fragments to prevent them from ligating to each other during the adapter ligation reaction by adding 12.5 μL of A-Tailing Mix. A corresponding single "T” nucleotide on the 3′ end of the adapter provides a complementary overhang for ligating the adapter to the fragment. Therefore 2.5 μL of Ligation Mix and 2.5 μL of the desired dual adapter were added. The products were purified and enriched by PCR (14 cycles) to create the final cDNA library. Library stock samples were diluted to 3 nM and samples with different indexes were pooled before clustering. The final sequencing Pool was diluted to 340 pM and loaded together with 0.6 μL of 2.5 nM PhiX Control v3 onto a SP flowcell (Illumina Inc., San Diego, CA, USA).

### RNA sequencing data analysis

Sequencing output was demultiplexed following the standard recommendations by the manufacturer and subsequent FastQ files were processed using a custom in-house pipeline for RNAseq analysis. Quality control was performed using FastQC [37], reads were then mapped using STAR [38] to the human reference genome GRCh38 and counts were obtained using RSEM [39]. Subsequent normalization of reads was performed using DESeq2 [40]. The deconvolution analysis was performed using the immunedeconv toolkit [41]. Visualization of all results was performed using ggplot2 [42]. The gene scores for interferon (IFN)-gamma [43] and tertiary lymphoid structures (TLS) [44] used in this study were computed using the gene set variation analysis algorithm [45]. As the number of samples in the study was considered low, we performed a further distribution analysis of the gene expression of our investigated set of gene expression values and compared the distribution to the overall expression pattern of all detected genes in each sample to determine whether we randomly picked arbitrary expression outliers. The gene expression for the set of genes investigated does not lie on the extreme borders of the overall transcript per million distribution of the samples (Supplementary Fig. 1).

### Statistical analyses

Statistical analyses were performed in R software version 3.6.3 for Windows. Decay-corrected radioactivity counts (Bq/mL) were processed to percentage injected activity (%IA), SUV, or organ-/tumor-to-plasma ratios. For organs and plasma, this data was parametric and reported as a mean with standard deviation. Tumor uptake was non-parametric and reported as a median with interquartile range (IQR). Exact *n* values and used statistical tests are reported at each figure if appropriate. A *p* value below 0.05 was considered statistically significant.

## Results

### Study procedures and patient demographics

**Fig. 1:**
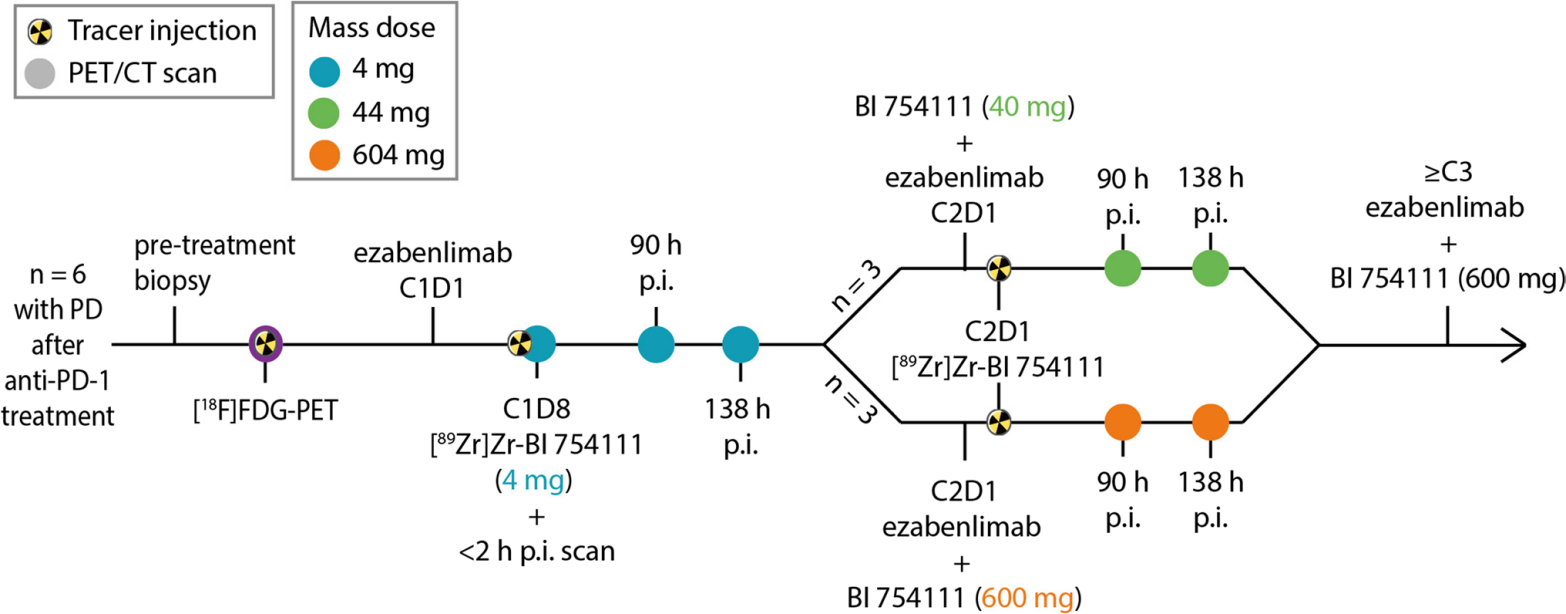
Study design. A schematic overview of study procedures is shown. Patients with progressive disease on previous anti-PD-1 therapy underwent a pre-treatment biopsy, received a [^18^F]FDG-PET scan, and restarted anti-PD-1 treatment with ezabenlimab 240 mg (C1D1). The radiolabeled anti-LAG-3 antibody [^89^Zr]Zr-BI 754111 was administered on C1D8 with a dose of 37 MBq and containing 4 mg of BI 754111. Scans were acquired < 2, 90, and 138 h p.i. Thereafter, on C2D1, 3 patients were administered 40 mg of unlabelled BI 754111 before a second tracer injection (reaching a total mass dose of 44 mg), and 3 patients were administered 600 mg of unlabeled BI 754111 before a second tracer injection (reaching a total mass dose of 604 mg). Tracer injection occurred within 1 h of administration of the unlabeled BI 754111, and scans were performed 90 and 138 h p.i. Venous blood samples were drawn at every scan time point. Patients continued on three-weekly cycles of ezabenlimab (240 mg) and BI 754111 (at 600 mg) till disease progression or unacceptable toxicity occurred. *anti-PD-1* anti-programmed death 1, *CT* computed tomography, *[*^*18*^*F]FDG-PET* 2-deoxy-2-[fluorine-18]fluoro-D-glucose-positron emmission tomography, *p.i.* post-injection

Between March 2019 and September 2020, 8 patients (5 patients with NSCLC and 3 patients with HNSCC) were included. Two patients started treatment, but ultimately did not undergo imaging procedures due to clinical deterioration and technical problems. Eventually, 4 patients with NSCLC and 2 patients with HNSCC completed imaging procedures. Patients were 66 years old (median, range, 53–77) and had an ECOG performance status of 0 (*N* = 1) or 1 (*N* = 5). At the time of inclusion, all patients had disease progression after an initial clinical benefit (of at least 3 months of stable disease) or response on an anti-PD-1-containing treatment regimen. Study procedures included a pre-treatment biopsy, baseline [^18^F]FDG PET, restart of anti-PD-1 therapy (with ezabenlimab, 240 mg, three-weekly cycles), and, thereafter, sequential [^89^Zr]Zr-BI 754111 PET imaging exploring three different mass doses of unlabeled BI 754111: 4 mg, 44 mg, and 604 mg (Fig. 1). After completion of imaging procedures, patients continued with three-weekly cycles of ezabenlimab (240 mg) and BI 754111 (600 mg) till disease progression or unacceptable toxicity occurred. Patient characteristics are detailed in Table 1.

**Table 1:** Characteristics of patients that completed imaging procedures (*n* = 6)

Characteristic	Values
Age	
Median (range)	66 (53–77)
Sex	
Male, *n* (%)	5 (83)
Female, *n* (%)	1 (17)
Histopathological diagnosis	
HNSCC, *n* (%)	2 (33)
Lung adenocarcinoma, *n* (%)	4 (66)
Involved metastatic sites	
Lymph nodes, *n* (%)	6 (100)
Lung, *n* (%)	1 (17)
Bone, *n* (%)	2 (33)
Breast, *n* (%)	1 (17)
Subcutaneous, *n* (%)	1 (17)
Subpleural, *n* (%)	1 (17)
Mediastinal, *n* (%)	1 (17)
Gastric wall, *n* (%)	1 (17)
ECOG performance score	
0, *n* (%)	1 (17)
1, *n* (%)	5 (83)
Previous chemotherapy, *n* (%)	3 (50)
Previous PD-1-containing treatment	
Anti-PD-1 monotherapy, *n* (%)	5 (83)
Anti-PD-1 + chemotherapy, *n* (%)	1 (17)
Best response to previous PD-1-containing treatment	
Stable disease (> 3 months), *n* (%)	3 (50)
Partial response, *n* (%)	3 (50)
Duration of previous PD-1-containing treatment	
Median months on treatment (range), *n* (%)	6.3 (3.7–27.3)

*HNSCC* head and neck squamous cell carcinoma, *ECOG* Eastern Cooperative Oncology Group, *PD-1* programmed death 1

### Pharmacokinetic analysis

**Fig. 2:**
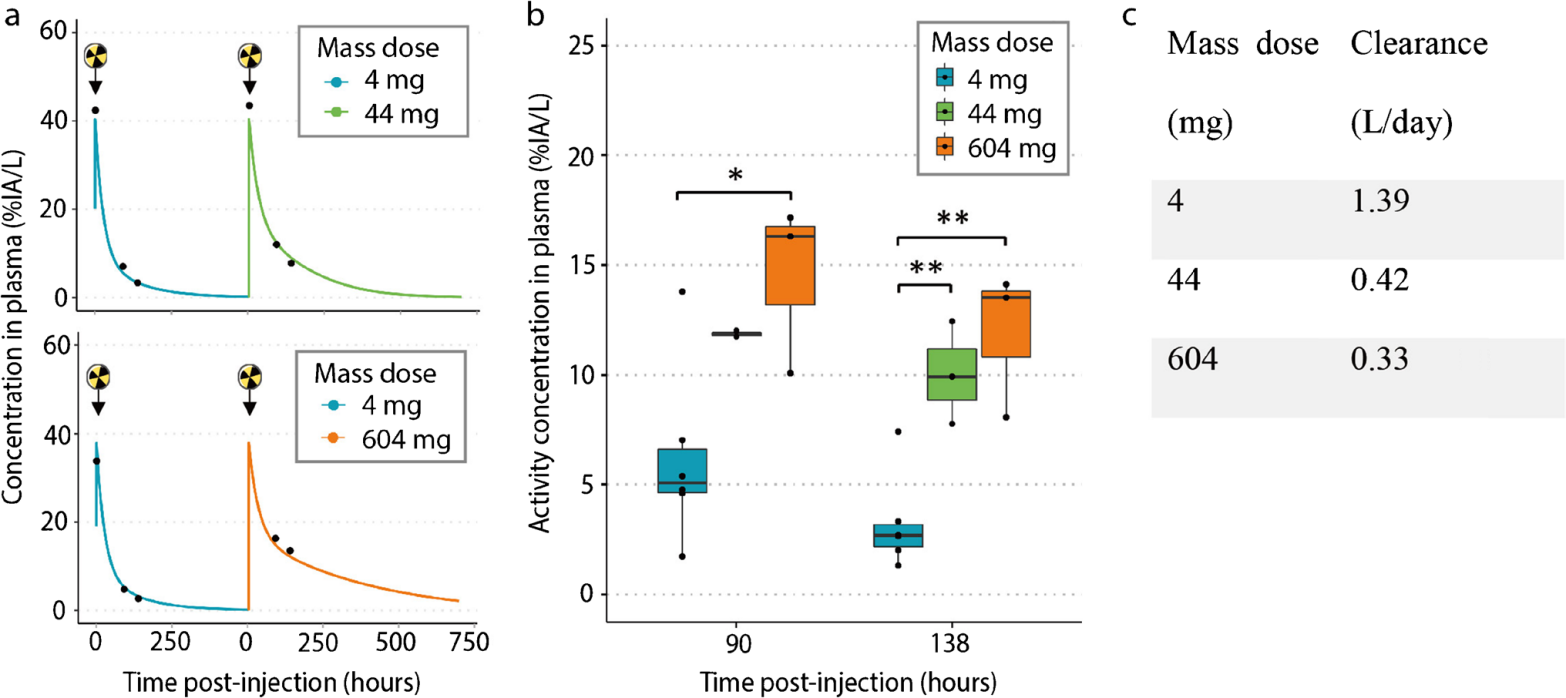
Clearance of the 44- and 604-mg mass doses was slower than that of the 4-mg mass dose. **a** The measured activity concentration in plasma (black dots) corresponded well to the popPK model from unlabelled BI 754111 (lines). Tracer injections are shown using black arrows and radioactivity signs. **b** The 44- and 604-mg mass doses resulted in higher activity concentrations in plasma than the 4-mg mass dose. One-way ANOVA (90 h p.i., F = 5.3, Df = 2; and 138 h p.i., F = 14.73, Df = 2), followed by Tukey HSD test was performed. For 90 h p.i.: *p* > 0.05 (4 mg vs 44 mg), *p* = 0.03 (4 mg vs 604 mg); and for 138 h p.i.: *p* = 0.01 (4 mg vs 44 mg) and *p* = 0.002 (4 mg vs 604 mg). Data comprises *n* = 6 for the 4-mg mass dose, *n =* 2 for the 44-mg mass dose 90 h p.i. and *n =* 3 138 h p.i. (one blood sample measurement failed 90 h p.i.), and *n* = 3 for the 604-mg mass dose. **c** Estimated clearances based on the popPK model of unlabeled BI 754111 differ between 4, 44, and 604 mass doses. Clearance corresponds to that at median *C*_max_ of the respective dose level. *%IA/L* percentage injected activity per L, *C*_max_ maximum plasma concentration, *Df* degrees of freedom; *HSD* honestly significant difference, *p.i.* post-injection, *popPK* population pharmacokinetic

After each [^89^Zr]Zr-BI 754111 PET scan, plasma samples were taken to measure ACs, expressed as %IA per L (%IA/L). The observed plasma ACs after administration of [^89^Zr]Zr-BI 754111 were in line with plasma concentrations predicted by a population pharmacokinetic (popPK) model for unlabeled BI 754111, suggesting that the ^89^Zr-labeling was stable and did not change the properties or plasma exposure of BI 754111 (Fig. 2a). This popPK model was based on data from a previous phase I study (NCT03156114); parameters are shown in Supplementary Table 1. Next, plasma ACs were assessed at the 4-mg, 44-mg, and 604-mg mass dose. In cycle 1, at the 4-mg mass dose, the mean plasma AC was 6.2 ± 4.1 %IA/L 90 h p.i., declining to 3.2 ± 2.2 %IA/L 138 h p.i. In cycle 2, at the 44-mg and 604-mg mass dose, plasma ACs were higher than those at the 4-mg mass dose: at 90 h p.i. 11.9 ± 0.2 %IA/L and 14.5 ± 3.9 %IA/L, and at 138 h p.i. 10.0 %IA/L and 11.9 %IA/L, respectively, demonstrating a difference in clearance between these doses as was also predicted by the popPK model (Figs. 2b and c).

### Biodistribution in healthy organs

**Fig. 3:**
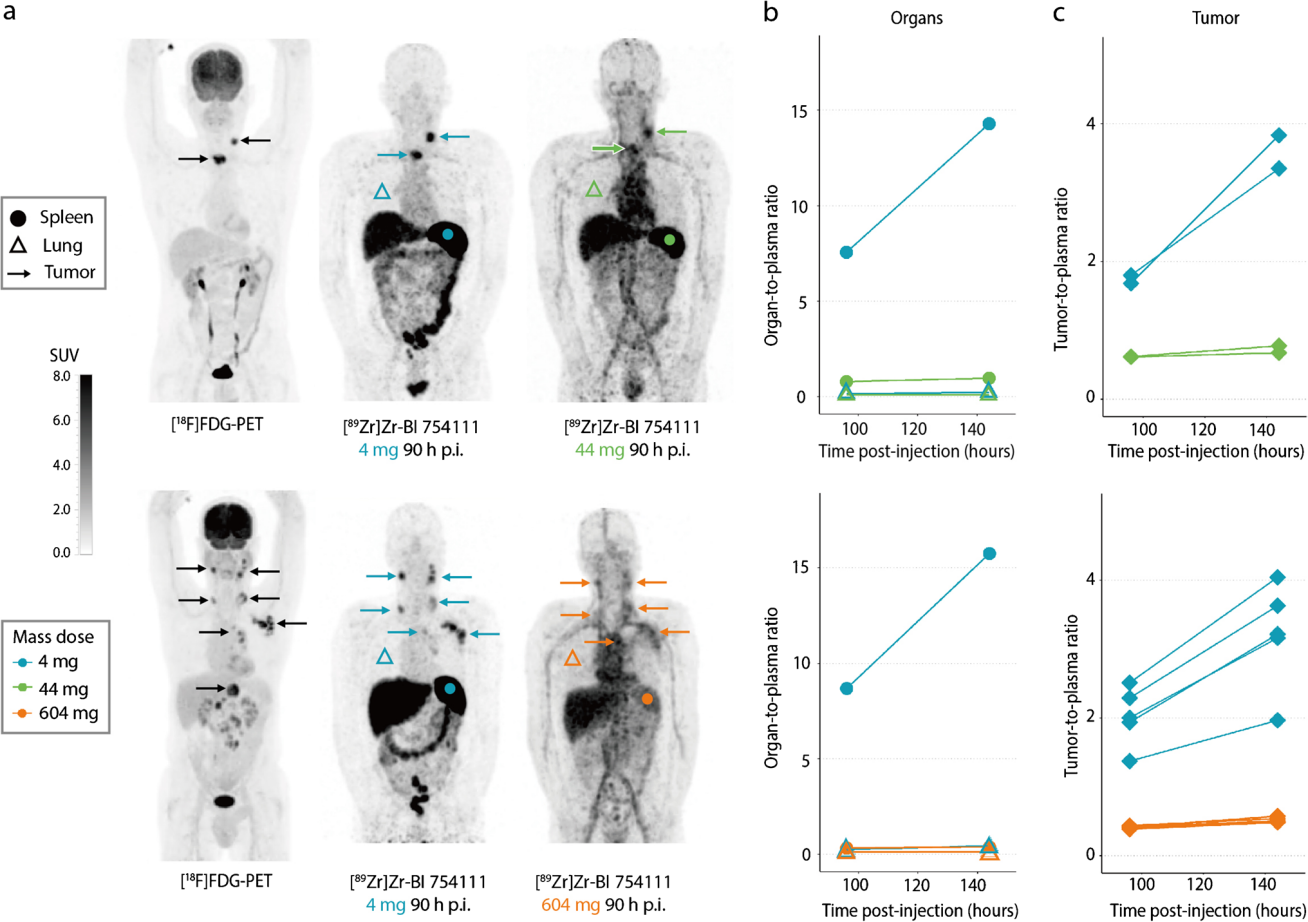
The biodistribution and tumor uptake of [^89^Zr]Zr-BI 754111. **a** Maximum intensity projections of [^18^F]FDG and [^89^Zr]Zr-BI 754111 PET scans of one patient from each dose cohort are shown: upper panel patient 10, HNSCC, 44-mg mass dose in cycle 2; lower panel patient 2, NSCLC, 604-mg mass dose in cycle 2. Favorable organ- and tumor-to-background contrast was seen at 4-mg mass dose. The higher doses, i.e., 44 mg and 604 mg, showed higher intravascular uptake, reducing the organ- and tumor-to-background ratios. Tumors are indicated by arrows, organs by symbol ((bullet) spleen, ((white triangle) lungs), and corresponding mass dose by color. **b** Uptake in spleen could be nearly saturated at the 44-mg dose and fully saturated at the 604-mg dose. Lung uptake did not change. Data on other organs can be found in Supplementary Table 2. **c** Uptake in different tumor lesions is variable and can be saturated by administering unlabeled BI 754111 pre-tracer injection, reaching mass doses of 44 mg and 604 mg. *[*^*18*^*F]FDG* 2-deoxy-2-[fluorine-18]fluoro-D-glucose, *HNSCC* head and neck squamous cell carcinoma, *NSCLC* non-small cell lung cancer, *PET* positron emission tomography, *p.i.* post-injection

PET images of the baseline [^18^F]FDG-PET, and two [^89^Zr]Zr-BI 754111 PET scans from one representative patient for each mass dose group, are presented in Fig. 3a. High uptake of [^89^Zr]Zr-BI 754111 was visible in the gall bladder and bowel, consistent with hepatobiliary clearance. To quantify organ uptake under different mass doses, the preferred uptake parameter was the organ-to-plasma ratio. For completeness, SUV values are available in the supplementary information. Organ-to-plasma ratios at the 4-mg mass dose (cycle 1) at 138 h p.i. revealed high uptake in spleen (mean 19.9 ± 14.9) and low uptake in lungs (mean 0.36 ± 0.16) (Fig. 3b; Supplementary Table 2). Increasing the mass dose by adding unlabeled BI 754111 in cycle 2 resulted in spleen-to-plasma ratios to drop to mean 1.55 ± 0.82 at the 44-mg mass dose, and to further decline to mean 0.47 ± 0.21 at the 604-mg mass dose, indicating saturation of target-specific binding sites in the spleen. A complete overview of organ-to-plasma ratios as well as SUVs can be found in Supplementary Table 2. To distinguish irreversible from reversible uptake, Patlak linearization was performed, demonstrating a net irreversible uptake component (*K*_*i*_) in the spleen at the 4-mg mass dose of 30.06 ± 11.17 μL g^−1^ h^−1^, which was partially saturated at the 44-mg mass dose (2.91 ± 0.79 μL g^−1^ h^−1^), and fully saturated at the 604-mg mass dose (0.67 ± 0.39 μL g^−1^ h^−1^), considering the baseline *K*_*i*_ values postulated by Jauw et al. (Supplementary Table 3) [32].

### Uptake in tumor lesions

**Fig. 4:**
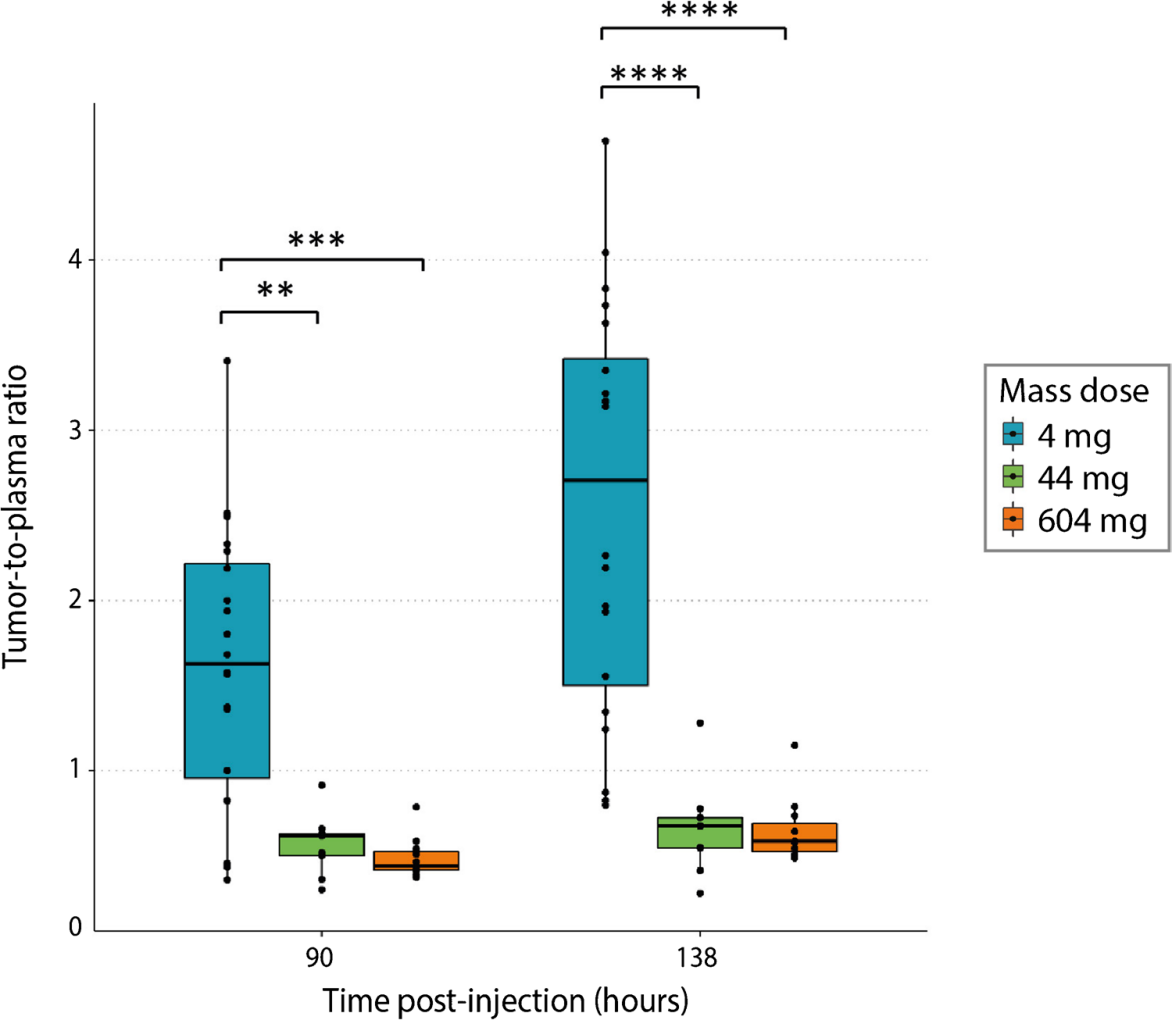
Tumor-to-plasma ratios of [^89^Zr]Zr-BI 754111 at different mass doses of BI 754111: tumor uptake of [^89^Zr]Zr-BI 754111 was highest at the 4-mg dose and was increasingly saturated at the 44-mg and 604-mg doses. Kruskall-Wallis with post hoc Wilcoxon rank-sum tests (two-sided) with Bonferroni correction were performed (****, *p* < 0.0001; ***, *p* < 0.001; **, *p* < 0.01, at 4-mg mass dose *n =* 20, at 44-mg mass dose *n =* 9, at 604-mg mass dose *n* = 11)

Patients underwent a baseline [^18^F]FDG PET and diagnostic CT scan to identify all tumor lesions with a diameter larger than 2 cm. Twenty tumor lesions (2–5 per patient), with an average tumor volume of 16.6 mL (range, 3.6–109.12 mL) and a mean SUV_max_ value of 9.01 ± 4.3, as assessed on [^18^F]FDG PET, were identified. Uptake of [^89^Zr]Zr-BI 754111 could be visualized in all 20 tumor lesions at the 4-mg dose showing a favorable tumor-to-background ratio (Fig. 3a). Tumor-to-plasma ratios at the 4-mg mass dose (in cycle 1) were 1.63 (IQR 0.37–2.89) 90 h p.i. and increased over time to a median of 2.70 (IQR 0.85–4.62) 138 h p.i. Adding unlabeled BI 754111 to reach mass doses of 44 mg and 604 mg (in cycle 2) significantly decreased tumor-to-plasma ratios 138 h p.i. to a median of 0.67 (IQR 0.50–0.85, *p* = 3.6*10^−6^) and 0.58 (IQR 0.42–0.75, *p* = 5*10^−7^), respectively (Figs. 3c and 4). Tumor-to-plasma ratios were not significantly different between the 44-mg and 604-mg doses (*p* = 0.88). Lymph node metastases, which potentially contain more/easily accessible LAG-3 expressing immune cells compared to other solid metastases, show higher uptake at 90 h p.i., but not at 138 h p.i. (Supplementary Fig. 2). SUV_peak_ values can be found in Supplementary Fig. 3. Additional PET images are shown in Supplementary Fig. 4.

### Correlation of tumor uptake with IHC and RNA sequencing

**Fig. 5:**
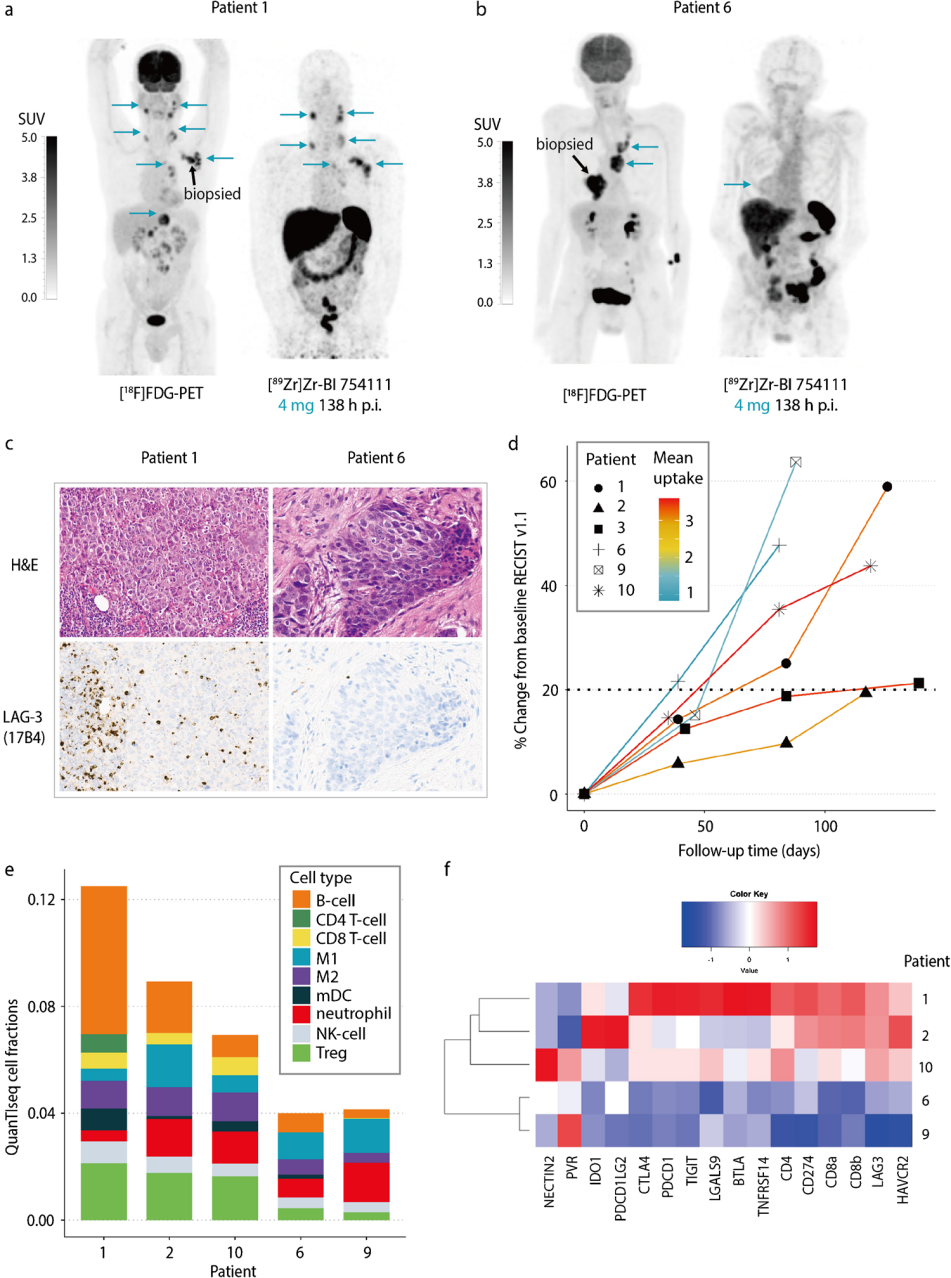
Correspondence of tumor uptake of [^89^Zr]Zr-BI 754111 with IHC and RNA expression. Uptake of [^89^Zr]Zr-BI 754111 was different between **a** patient 1 and **b** patient 6 (representative of patients with high and low tumor uptake, respectively). Tumor lesions are indicated with blue arrows and biopsied lesions are indicated with black ones. **c** Corresponding H&E and LAG-3 IHC stainings are shown for biopsied lesions (patient 1: 5%, patient 6: <1% LAG-3+ imunne cells per tumor area). **d** Mean uptake of [^89^Zr]Zr-BI 754111 in patients 6 and 9 was lower than for patients 1, 2, 3, and 10. **e** RNA-sequencing revealed that the low uptake corresponded to a low abundancy of immune cells, and **f** low expression of T-cell related genes. Note that for patient 3 a biopsy could not be obtained. *CD4 T-cell* conventional (non-regulatory) CD4 T-cell, *[*^*18*^*F]FDG-PET* 2-deoxy-2-[fluorine-18]fluoro-D-glucose-positron emmission tomography, *H&E* hematoxylin & eosin, *IHC* immunohistochemistry, *M1* M1-like macrophage, *M2* M2-like macrophage, *mDC* myeloid derived dendritic cell; *NK* natural killer cell, *p.i.* post-injection, *SUV* standardized uptake value, *Treg* T-regulatory cell

Tumor-to-plasma ratios of [^89^Zr]Zr-BI 754111 at the 4-mg dose were used for correlation with translational analyses, to minimalize the influence of saturation effects. Pre-treatment biopsies were successfully collected for all patients, except for patient 3. Individual outcomes and corresponding patient characteristics are available in Table 2. Tumor uptake was heterogeneous at the 4-mg dose 138 h p.i.: 2 patients were at the low end of the spectrum (mean tumor-to-plasma ratio 0.8 and 1.4 for patients 6 and 9, respectively), and 4 patients (1, 2, 3, and 10) demonstrated a higher mean tumor uptake (mean tumor-to-plasma ratio ≥ 3.0 for all patients). PET scans for patients 1 and 6 are shown as examples in Fig. 5a and b. Immunohistochemical staining of biopsied lesions confirmed low percentages of LAG-3 positive immune cells per tumor area for patients 6 and 9 (≤ 1% and 5%, respectively), as compared to patients 1, 2, and 10 (10%, 5%, 30%, respectively), supporting the notion that low mean tumor uptake of [^89^Zr]Zr-BI 754111 corresponds to low abundancy of (LAG-3+) immune cells in the TME (Fig. 5c). Moreover, the mean low uptake of patients 6 and 9 coincided with a slightly faster tumor growth as demonstrated by the 48% and 64% increase in tumor volume at 12 weeks (Fig. 5d). All patients ultimately progressed within 18 weeks. Next, RNA-sequencing on baseline tumor biopsies was performed and cell deconvolution using quanTIseq revealed that the tumor lesions of patients 6 and 9 were practically void of conventional effector CD4 and CD8 T-cells and T-cell related mRNAs (Fig. 5e), whereas the other patients (patients 1, 2, and 10) did show a clear activated effector T-cell signature with relatively higher expression of immune checkpoints, including LAG-3 (Fig. 5f). Besides evidence of CD4 and CD8 T-cell presence in tumor biopsies from patients 1, 2, and 10, also a relative abundance of Tregs and B-cells was noted (Fig. 5e). A similar clustering of patients was found when looking at an RNA signature related to T-cell infiltration and T-cell priming in TLS, which have shown to have prognostic relevance in various solid tumor types including melanoma [44], oral squamous cell carcinoma [46], and NSCLC [47] (Supplementary Fig. 5). The presence of an IFN-gamma signature confirmed the activated state and apparent functionality of the detected T cells (Supplementary Fig. 6).

**Table 2:** Clinical and translational outcomes per study subject included in imaging procedures

	1	2	3	6	9	10
Histopathological diagnosis	NSCLC	NSCLC	NSCLC	HNSCC	NSCLC	HNSCC
Mass dose in cycle 2 (mg)	604	604	604	44	44	44
Mean tumor-to-plasma ratio 138 h p.i.	3.20	3.02	3.44	0.83	1.43	3.59
PFS (days)	126	188	139	81	88	119
LAG-3 immunoreactivity (% positive cells per tumor area)	10	5	NA	<1	5	30

*HNSCC* head and neck squamous cell carcinoma, *LAG-3* lymphocyte-activation gene 3, *NA* not available, *NSCLC* non-small cell lung cancer, *PFS* progression-free survival, *p.i.* post-injection

### Clinical outcome and adverse events

Treatment was well tolerated; adverse events are shown in Supplementary Table 4 and are in line with the safety profile of checkpoint inhibitors. No objective responses were observed; the best clinical outcome was stable disease and was observed in 4 out of 8 patients.

## Discussion

To the best of our knowledge, this is the first PET imaging study targeting LAG-3 in cancer patients. Even though the amount of LAG-3 targets in the TME is reportedly low [19], the PET signal in tumors at the 4-mg mass dose was clearly visible and target specific, two important requirements for a potential predictive imaging biomarker. At this dose, high uptake of [^89^Zr]Zr-BI 754111 was also noted in the spleen, again surpassing expectations based on reported protein expression of LAG-3 in the spleen [19, 20].

The discrepancy between the reported low protein expression of LAG-3 and the observed high PET uptake of [^89^Zr]Zr-BI 754111 may have several explanations. Firstly, all patients were restarted on ezabenlimab (anti-PD-1 mAb) treatment 8 days before tracer administration, which may have resulted in a (transient) increase in LAG-3 expression. Secondly, the intensity of the LAG-3 IHC signal is often not quantified beyond a categorical variable such as −/+/++, making IHC a less than optimal method for LAG-3 quantification and comparison with PET uptake. In addition, a tumor biopsy only contains a small fraction (~ 0.1%) of the tumor tissue. Thirdly, upon binding of [^89^Zr]Zr-BI 754111 to LAG-3, the mAb-LAG-3 complex internalizes and becomes degraded. However, ^89^Zr is a residualizing isotope that accumulates in the cell over time, representing a compounded signal rather than the target expression at a fixed point in time. This is especially of interest for targets like LAG-3, since new LAG-3 receptors can be synthesized at a rate of ~ 10% per hour in T cells [21], and thus, this process can result in a considerably enhanced PET tracer signal even when the target protein expression is low.

For a potential predictive imaging biomarker to be successful, the PET signal not only needs to be quantifiable, but also needs to be target specific. To this end, unlabeled BI 754111 mAb was administered prior to [^89^Zr]Zr-BI 754111 injection—thereby increasing the mass dose of the tracer. For the spleen, this resulted in partial saturation of the spleen-to-plasma ratio at the 44-mg dose, and complete saturation at the 604-mg dose, demonstrating that this signal is indeed target specific. These results were confirmed by Patlak analyses—a method to isolate irreversible uptake from reversible uptake [32]—which also demonstrated that for the spleen the irreversible uptake was completely saturated at the 604-mg dose. For tumor lesions, increasing the mass dose also resulted in a decrease in tumor-to-plasma ratios, which demonstrates target specificity, and is consistent with LAG-3 saturation at these doses. Since BI 754111 (combined with ezabenlimab) is a three-weekly treatment and the latest time-point in this study is 138 h p.i. (~ 6 days), saturation during the full 3 weeks cannot be assessed. However, employing ^89^Zr-immuno-PET data in such a way can potentially help inform decisions on an RP2D in the future, especially for mAb therapies where due to the lack of acute toxicities an RP2D dose is hard to establish. As a side note, PET imaging with anti-LAG-3 can be employed as a pharmacodynamic biomarker to detect upregulation of LAG-3 in response to different immunotherapeutic strategies, revealing potential mechanisms of resistance or uncovering new potential applications of anti-LAG-3 therapy.

A requirement that is becoming increasingly more relevant for potentially predictive imaging biomarkers, is the degree of corroboration by tissue-based analyses. RNA-sequencing of pre-treatment biopsies identified two patients (6 and 9) with a low number of immune cells in the tumor (i.e., immune desert tumors) at baseline, which corresponded to low tumor uptake of [^89^Zr]Zr-BI 754111 at the 4-mg dose. Higher tumor uptake of [^89^Zr]Zr-BI 754111 observed in patients 1, 2, and 10 coincided with a higher CD4 and CD8 T-cell content and an activated T-cell signature at baseline, including relatively high expression levels of LAG-3 and other immune checkpoints, as well as evidence of increased Tregs and B-cells. The concomitant presence of a TLS signature fits with the (marginally) improved clinical outcome in these patients. Although the sample size should be expanded for definitive conclusions, these data support the PET imaging results and are a first attempt at using RNA-sequencing to substantiate PET tracer uptake findings.

In parallel, four other anti-LAG-3 radiotracers are currently under development: three anti-LAG-3 nanobody-based tracers, two of which have shown promising results in pre-clinical studies [48–50], and a third which is currently recruiting up to 50 patients with solid tumors (NCT05346276). The fourth is another ^89^Zr-labelled anti-LAG-3 mAb ([^89^Zr]Zr-DFO-REGN3767) that is under investigation in two different clinical trials focusing on the biodistribution and pharmacokinetics of this tracer (NCT04706715, NCT04566978). The results of these studies are eagerly awaited, specifically in the case of the nanobodies, as they have faster kinetics than [^89^Zr]Zr-BI 754111, which will make for an interesting comparison to the current study. The next step for a potential predictive imaging biomarker is a larger study that could test the correlation between PET tumor uptake and response to treatment. However, expanding the sample size in these types of studies is often limited by factors such as radiation burden and scan duration. These hurdles might be overcome by the use of the upcoming new generation of long-axial field-of-view PET scanners with ultra-high sensitivity (i.e., “total-body PET”), which substantially reduce scan time and radiation burden for patients, next to a strongly improved image quality [51].

## Conclusions

In this study, we show that LAG-3 is a feasible target for generating a predictive imaging biomarker, with [^89^Zr]Zr-BI 754111 demonstrating a favorable tumor-to-background uptake, target specificity, and correlation with the immune status of the tumor biopsies at baseline. These favorable technical and biological characteristics support further investigation of [^89^Zr]Zr-BI 754111 and other LAG-3-directed PET tracers for the development of predictive imaging biomarkers for anti-LAG-3 therapy.

## Supplementary information

Supplementary file 1

## Data Availability

Data to support the findings in this study are included in the manuscript or its supplementary information. Additional data can be made available upon reasonable request from the corresponding author (c.menke@amsterdamumc.nl).
