# Supplementary material for: 89Zr-immuno-PET using the anti-LAG-3 tracer [89Zr]Zr-BI 754111: demonstrating target specific binding in NSCLC and HNSCC
Source: Eur J Nucl Med Mol Imaging. 2023 Mar 2;50(7):2068–80. doi: 10.1007/s00259-023-06164-w (PMC10199858; doi:10.1007/s00259-023-06164-w)
Supplement: Supplementary file 1 [file 259_2023_6164_MOESM1_ESM.docx]

**Supplementary information**

**Tracer synthesis and quality control**

BI 754111 (5 mg, 20 mg/mL) was diluted to 5 mg/mL with 0.9% NaCl and the pH of the solution adjusted to 8.9–9.1 with 0.1 M Na_2_CO_3_ after which the solution was added to benzyl isothiocyanate-DFO (DFO-Bz-NCS) (3 equivalents in dimethyl sulfoxide [DMSO], 100 nmol, 20 µL of 5 mM) and reacted for 30 min at 37°C. DFO-Bz-NCS-BI754111 was purified on a pyrogen-free PD10 column to remove unreacted chelator using 25 mM NaOAc/240 mM Trehalose/0.67 mM Methionine/0.04% w/v Tween-20 pH 5.5 as eluent. This purified DFO-Bz-NCS-BI754111 was then labeled with Zr-89. To this end, the required amount of ^89^Zr in 1 M oxalic acid (Perkin Elmer, Boston, MA, USA) was supplemented to 0.2 mL with 1 M oxalic acid, followed by the addition of 90 µL 2 M Na_2_CO_3_ to neutralize the solution. Hereafter, 1 mL 0.5 M Hepes was added, followed by 0.71 mL DFO-Bz-NCS-BI 754111 (~2.3 mg). After labeling (60 min), [^89^Zr]Zr-DFO-Bz-NCS-BI 754111 was purified on a pyrogen free PD10 column and eluted in 25 mM NaOAc/240 mM Trehalose / 0.67 mM Methionine/0.04% w/v Tween-20 pH 5.5. Chemical modifications to generate [^89^Zr]Zr-DFO-Bz-NCS- BI 754111 had no impact on BI 754111 binding affinity to lymphocyte-activation gene 3 (LAG-3) as was demonstrated by Surface Plasma Resonance based assays (data not shown).

The product was formulated to arrive at an injection dose of 37 MBq – 4 mg – 20 mL [^89^Zr]Zr-BI 754111. The pH of the product was 5.49 ± 0.02. The mean radiochemical purity as assessed by spin filter was 99.3% ± 0.5%. To this end, 4 µL of product was diluted with 96 µL eluent (5% DMSO and 95% 50 mM NaOAc + 200 mM sucrose buffer) and applied on a microcon-30 centrifugal filter unit (Ultracel YM-30, regenerated cellulose, 30 kDa cut-off, Merck Millipore, Burlington, MA, USA). The solution was spun down for 7 min at 14,000 rpm (Eppendorf 5430). The filter was washed twice with 100 µL eluent and spun down at 14,000 rpm for 7 min after each wash step. The filtrate contained free ^89^Zr/^89^Zr-DFO, while the radiolabeled mAb was left on the filter. The mean radiochemical purity was 99.7 ± 0.6% and the mean protein integrity was 98.9% ± 0.2% as determined by size exclusion high-performance liquid chromatography using a superdex 200 10/30 GL increase size exclusion column (GE healthcare Life sciences, Piscataway, NJ, USA) including a guard column using a mixture of 0.05 M sodium phosphate, 0.15 M sodium chloride (pH 6.8), and 0.01 M NaN_3_ as the eluent at a flow rate of 0.75 mL/min. The mean immune reactive fraction as assessed by a LAG-3 binding assay was 78.6% ± 4.1%. Sterility of each [^89^Zr]Zr-BI 754111 batch was assured by performing a media fill immediately after final filter sterilization of each batch. These procedures resulted in a sterile final product with endotoxin levels <0.2 EU/mL.

**Supplementary Table 1.1.** Intended patient distribution across dose groups in pharmacokinetic model development

| Dose (mg) | *n* |
| --- | --- |
| 4 | 4 |
| 20 | 9 |
| 80 | 9 |
| 200 | 9 |
| 400 | 9 |
| 600 | 9 |

**Supplementary Table 1.2.** Population pharmacokinetic model parameters

| Parameter | Value | Units |
| --- | --- | --- |
| Fixed effect parameters | | |
| Cl | 0.0122 | L/h |
| Vc | 3.40 | L |
| Vp | 2.25 | L |
| Q | 0.0353 | L/h |
| Clsaturable | 0.0489 | L/h |
| C50 | 216 | ug/L |
| Between subject variability | | |
| ω2 | Cl | 0.149 |
| ω2 | Vc | 0.0473 |
| Residual error | | |
| Proportional (CV) | 10.0E+00 | % |

*Cl* clearance, *CV* coefficient of variation, *Q* intercompartmental clearance, *Vc* central volume of distribution, *Vp* peripheral volume of distribution

**Supplementary Table 2.1.** Organ-to-plasma ratios of [^89^Zr]Zr-BI 754111

| Organ | Dose | Time p.i. (h) | *n* | Mean | SD | Organ | Dose | Time p.i. (h) | *n* | Mean | SD |
| --- | --- | --- | --- | --- | --- | --- | --- | --- | --- | --- | --- |
| Bone marrow | 4 | 2 | 3 | 0.12 | 0.02 | Liver | 4 | 2 | 3 | 0.25 | 0.03 |
|  | 4 | 96 | 6 | 0.84 | 0.69 |  | 4 | 96 | 6 | 2.42 | 1.82 |
|  | 4 | 144 | 6 | 1.36 | 0.67 |  | 4 | 144 | 6 | 4.30 | 2.09 |
|  | 44 | 96 | 3 | 0.30 | 0.11 |  | 44 | 96 | 3 | 0.79 | 0.31 |
|  | 44 | 144 | 2 | 0.35 | 0.07 |  | 44 | 144 | 2 | 1.26 | 0.93 |
|  | 604 | 96 | 3 | 0.16 | 0.04 |  | 604 | 96 | 3 | 0.45 | 0.02 |
|  | 604 | 144 | 3 | 0.18 | 0.02 |  | 604 | 144 | 3 | 0.57 | 0.03 |
| Brain | 4 | 2 | 3 | 0.03 | 0.00 | Lung | 4 | 2 | 3 | 0.08 | 0.00 |
|  | 4 | 96 | 6 | 0.06 | 0.02 |  | 4 | 96 | 6 | 0.23 | 0.12 |
|  | 4 | 144 | 6 | 0.08 | 0.05 |  | 4 | 144 | 6 | 0.36 | 0.16 |
|  | 44 | 96 | 3 | 0.05 | 0.02 |  | 44 | 96 | 3 | 0.11 | 0.02 |
|  | 44 | 144 | 2 | 0.05 | 0.03 |  | 44 | 144 | 2 | 0.12 | 0.01 |
|  | 604 | 96 | 3 | 0.04 | 0.01 |  | 604 | 96 | 3 | 0.13 | 0.00 |
|  | 604 | 144 | 3 | 0.05 | 0.02 |  | 604 | 144 | 3 | 0.15 | 0.01 |
| Kidney | 4 | 2 | 3 | 0.12 | 0.02 | Spleen | 4 | 2 | 3 | 0.31 | 0.17 |
|  | 4 | 96 | 6 | 0.82 | 0.46 |  | 4 | 96 | 6 | 11.22 | 11.82 |
|  | 4 | 144 | 6 | 1.33 | 0.47 |  | 4 | 144 | 6 | 19.93 | 14.89 |
|  | 44 | 96 | 3 | 0.38 | 0.10 |  | 44 | 96 | 3 | 0.92 | 0.21 |
|  | 44 | 144 | 2 | 0.44 | 0.20 |  | 44 | 144 | 2 | 1.55 | 0.82 |
|  | 604 | 96 | 3 | 0.28 | 0.03 |  | 604 | 96 | 3 | 0.41 | 0.16 |
|  | 604 | 144 | 3 | 0.32 | 0.05 |  | 604 | 144 | 3 | 0.47 | 0.21 |

*p.i.* post-injection, *SD* standard deviation

**Supplementary Table 2.2.** Organ SUVmean uptake of [^89^Zr]Zr-BI 754111

| Organ | Dose | Time p.i. (h) | *n* | Mean | SD | Organ | Dose | Time p.i. (h) | *n* | Mean | SD |
| --- | --- | --- | --- | --- | --- | --- | --- | --- | --- | --- | --- |
| Bone  marrow | 4 | 2 | 3 | 2.73 | 0.06 | Liver | 4 | 2 | 3 | 5.73 | 0.20 |
|  | 4 | 96 | 6 | 2.71 | 0.76 |  | 4 | 96 | 6 | 7.48 | 1.99 |
|  | 4 | 144 | 6 | 2.59 | 0.73 |  | 4 | 144 | 6 | 8.00 | 1.87 |
|  | 44 | 96 | 3 | 2.20 | 0.47 |  | 44 | 96 | 3 | 5.81 | 1.91 |
|  | 44 | 144 | 2 | 2.32 | 0.58 |  | 44 | 144 | 2 | 7.30 | 2.50 |
|  | 604 | 96 | 3 | 1.80 | 0.24 |  | 604 | 96 | 3 | 5.25 | 0.75 |
|  | 604 | 144 | 3 | 1.67 | 0.04 |  | 604 | 144 | 3 | 5.43 | 0.82 |
| Brain | 4 | 2 | 3 | 0.60 | 0.10 | Lung | 4 | 2 | 3 | 1.88 | 0.33 |
|  | 4 | 96 | 6 | 0.24 | 0.11 |  | 4 | 96 | 6 | 0.77 | 0.30 |
|  | 4 | 144 | 6 | 0.17 | 0.10 |  | 4 | 144 | 6 | 0.68 | 0.16 |
|  | 44 | 96 | 3 | 0.34 | 0.09 |  | 44 | 96 | 3 | 0.81 | 0.23 |
|  | 44 | 144 | 2 | 0.30 | 0.04 |  | 44 | 144 | 2 | 0.82 | 0.41 |
|  | 604 | 96 | 3 | 0.48 | 0.18 |  | 604 | 96 | 3 | 1.52 | 0.28 |
|  | 604 | 144 | 3 | 0.46 | 0.21 |  | 604 | 144 | 3 | 1.43 | 0.25 |
| Kidney | 4 | 2 | 3 | 2.73 | 0.12 | Spleen | 4 | 2 | 3 | 6.84 | 3.15 |
|  | 4 | 96 | 6 | 2.73 | 0.70 |  | 4 | 96 | 6 | 32.33 | 15.37 |
|  | 4 | 144 | 6 | 2.58 | 0.48 |  | 4 | 144 | 6 | 36.12 | 15.84 |
|  | 44 | 96 | 3 | 2.84 | 0.52 |  | 44 | 96 | 3 | 7.12 | 2.51 |
|  | 44 | 144 | 2 | 2.76 | 0.01 |  | 44 | 144 | 2 | 9.54 | 0.78 |
|  | 604 | 96 | 3 | 3.23 | 0.15 |  | 604 | 96 | 3 | 4.65 | 1.26 |
|  | 604 | 144 | 3 | 3.01 | 0.09 |  | 604 | 144 | 3 | 4.30 | 1.23 |

*p.i.* post-injection, *SD* standard deviation, *SUV* standardized uptake value

**Supplementary Table 3.** Net irreversible uptake (*K_i_*) of [^89^Zr]Zr-BI 754111 presented as µL g^−1^ min^−1^ with SD in organs at different mass doses. Baseline *K_i_* values, based on Patlak analysis of monoclonal antibodies in organs without target expression, are adapted from Jauw et al. for comparison (1)

| Tissue | 4 mg mass dose  *K_i_* ± SD | 40 mg mass dose  *K_i_* ± SD | 604 mg mass dose  *K_i_* ± SD | Baseline  *K_i_* median (IQR) (1) |
| --- | --- | --- | --- | --- |
| Bone marrow | 1.95 ± 0.84 | 0.58 ± 0.13 | 0.27 ± 0.12 | NA |
| Brain | 0.05 ± 0.06 | 0.01 ± 0.030 | 0.07 ± 0.04 | NA |
| Kidney | 1.65 ± 0.58 | 0.46 ± 0.06 | 0.47 ± 0.13 | 0.7 (0.4–1.3) |
| Liver | 6.57 ± 2.00 | 2.30 ± 0.81 | 1.24 ± 0.16 | 1.1 (0.8–2.1) |
| Lung | 0.41 ± 0.22 | 0.14 ± 0.09 | 0.23 ± 0.03 | 0.2 (0.1–0.3) |
| Spleen | 30.06 ± 11.17 | 2.91 ± 0.79 | 0.67 ± 0.39 | 0.5 (0.3–0.7) |

*IQR* interquartile range, *K*_i_ net irreversible uptake component, *NA* not available, *SD* standard deviation

**Supplementary Table 4.** Treatment-emergent AEs by system organ class, preferred term, and maximum CTCAE grade for all included patients (*n* = 8)

| System organ class | Total number (percentage) of patients | | | |
| --- | --- | --- | --- | --- |
| Preferred term | **All grades** | **Grade 1** | **Grade 2** | **Grade 3** |
|  | ***n* (%)** | ***n* (%)** | ***n* (%)** | ***n* (%)** |
| Number of patients | 8 (100.0) | 8 (100.0) | 8 (100.0) | 8 (100.0) |
| Total with AEs | 7 (87.5) | 2 (25.0) | 2 (25.0) | 3 (37.5) |
| General disorders and administration site conditions | 5 (62.5) | 1 (12.5) | 1 (12.5) | 3 (37.5) |
| Pyrexia | 1 (12.5) | 0 | 0 | 1 (12.5) |
| Fatigue | 2 (25.0) | 1 (12.5) | 1 (12.5) | 0 |
| Malaise | 2 (25.0) | 0 | 0 | 2 (25.0) |
| Gastrointestinal disorders | 2 (25.0) | 0 | 2 (25.0) | 0 |
| Diarrhea | 1 (12.5) | 0 | 1 (12.5) | 0 |
| Food poisoning | 1 (12.5) | 0 | 1 (12.5) | 0 |
| Gastric hemorrhage | 1 (12.5) | 0 | 1 (12.5) | 0 |
| Musculoskeletal and connective tissue disorders | 2 (25.0) | 1 (12.5) | 0 | 1 (12.5) |
| Arthralgia | 1 (12.5) | 0 | 0 | 1 (12.5) |
| Musculoskeletal pain | 1 (12.5) | 0 | 0 | 1 (12.5) |
| Myalgia | 1 (12.5) | 0 | 0 | 1 (12.5) |
| Osteoarthritis | 1 (12.5) | 0 | 0 | 1 (12.5) |
| Pain in extremity | 1 (12.5) | 1 (12.5) | 0 | 0 |
| Respiratory, thoracic, and mediastinal disorders | 2 (25.0) | 1 (12.5) | 0 | 1 (12.5) |
| Chronic obstructive pulmonary disease | 1 (12.5) | 0 | 0 | 1 (12.5) |
| Dyspnea | 1 (12.5) | 1 (12.5) | 0 | 0 |
| Pharyngeal hemorrhage | 1 (12.5) | 1 (12.5) | 0 | 0 |
| Skin and subcutaneous tissue disorders | 2 (25.0) | 0 | 1 (12.5) | 1 (12.5) |
| Dry skin | 1 (12.5) | 0 | 1 (12.5) | 0 |
| Rash macular | 1 (12.5) | 0 | 0 | 1 (12.5) |
| Neoplasms benign, malignant, and unspecified (including cysts and polyps) | 2 (25.0) | 0 | 0 | 2 (25.0) |
| Metastases to bone | 1 (12.5) | 0 | 0 | 1 (12.5) |
| Metastases to central nervous system | 1 (12.5) | 0 | 0 | 1 (12.5) |

*AEs* adverse events, *CTCAE* Common Terminology Criteria for Adverse Events


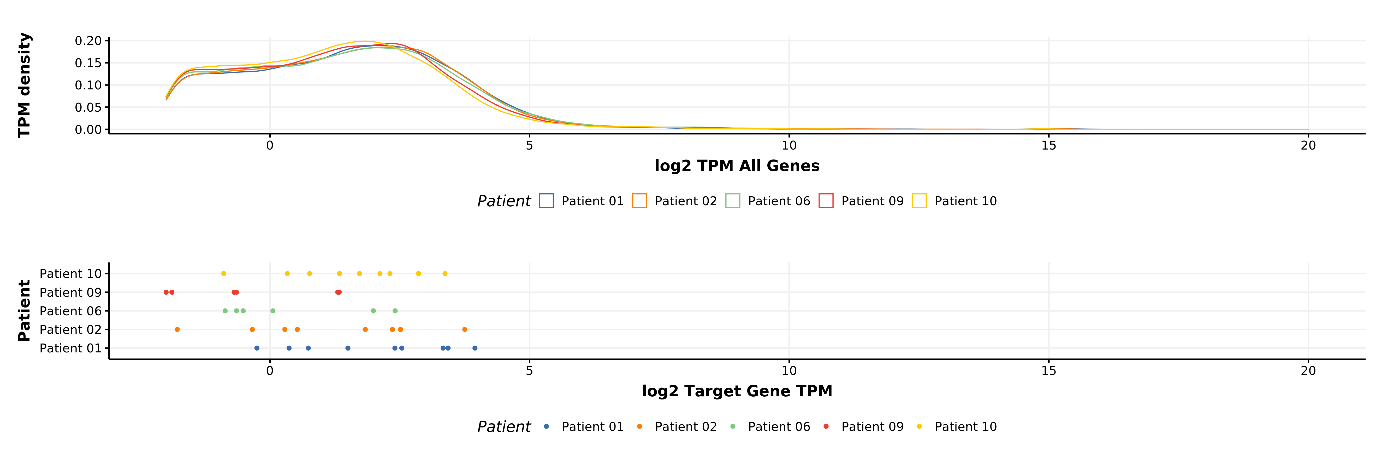


**Supplementary Fig. 1** RNA check gene frequencies. Comparative analysis of overall log2 TPM gene expression density (top) against the expression of our selected set of 15 genes per patient (bottom). As can be observed, the gene expression for the set of genes investigated does not lie on the extreme borders of the overall TPM distribution of the samples. Furthermore, the expression distribution of all investigated patient samples resembles each other, particularly pointing out that there is technical consistency across samples. *TPM* transcript per million


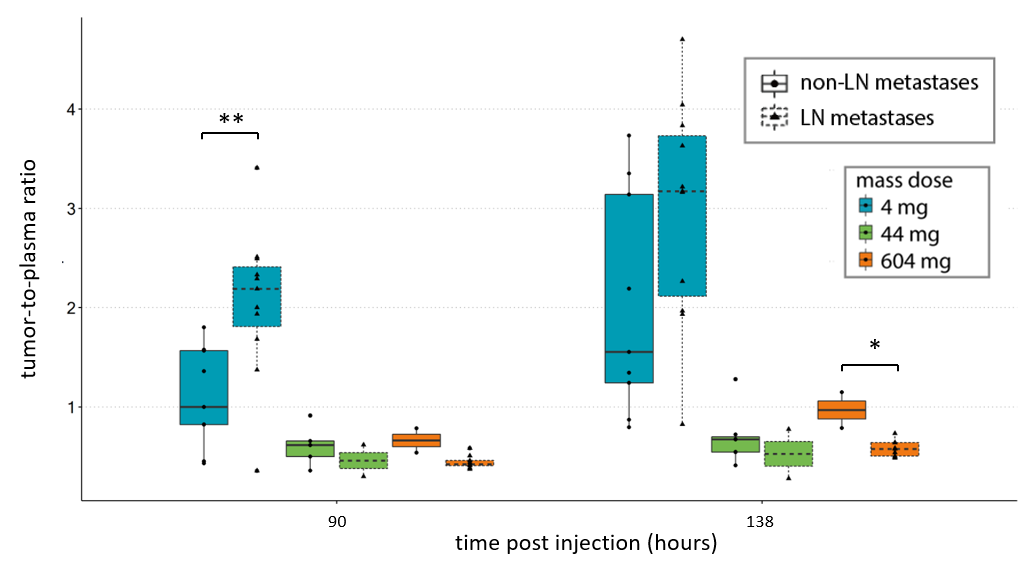
**Supplementary Fig. 2** [^89^Zr]Zr-BI 754111 tumor-to-plasma ratios for LN metastases versus non-LN metastases (i.e., solid tumors). LN metastases have a slightly higher tumor-to-plasma ratio at 90 h p.i. only for the 4 mg dose, but are not significantly different from non-LN metastases at 138 h p.i.. Non-LN metastases include soft tissue, bone, and lung metastases. Kruskall-Wallis with post-hoc Wilcoxon rank-sum tests (two-sided) with Bonferroni correction were performed (**, *p* < 0.01; *, *p* < 0.05, *n* for non-LN vs. LN metastases: at 4 mg mass dose *n =* 11 vs. *n =* 9, at the 44 mg mass dose *n =* 7 vs. *n =* 2, and at the 604 mg mass dose *n =* 2 vs. *n =* 9). *LN* lymph node, *p.i.* post-injection


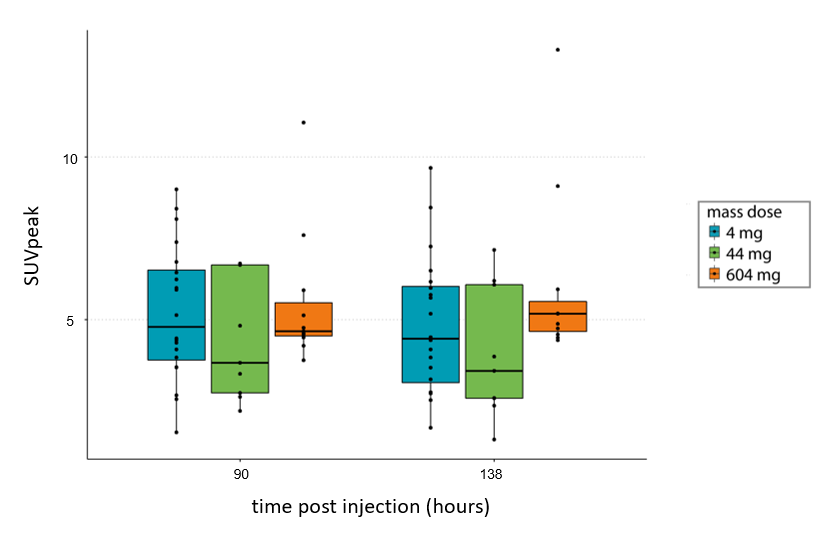


**Supplementary Fig. 3** SUVpeak tumor uptake of [^89^Zr]Zr-BI 754111. Tumor uptake of [^89^Zr]Zr-BI 754111, expressed as SUVpeak, did not differ between the 4 mg mass dose compared to the 44 mg and 604 mg mass doses (Kruskal-Wallis tests were performed, for the 4 mg dose *n* = 20, for the 44 mg mass dose *n* = 9, and for the 604 mg mass dose *n* = 11, *p* > 0.05). *SUV* standardized uptake value


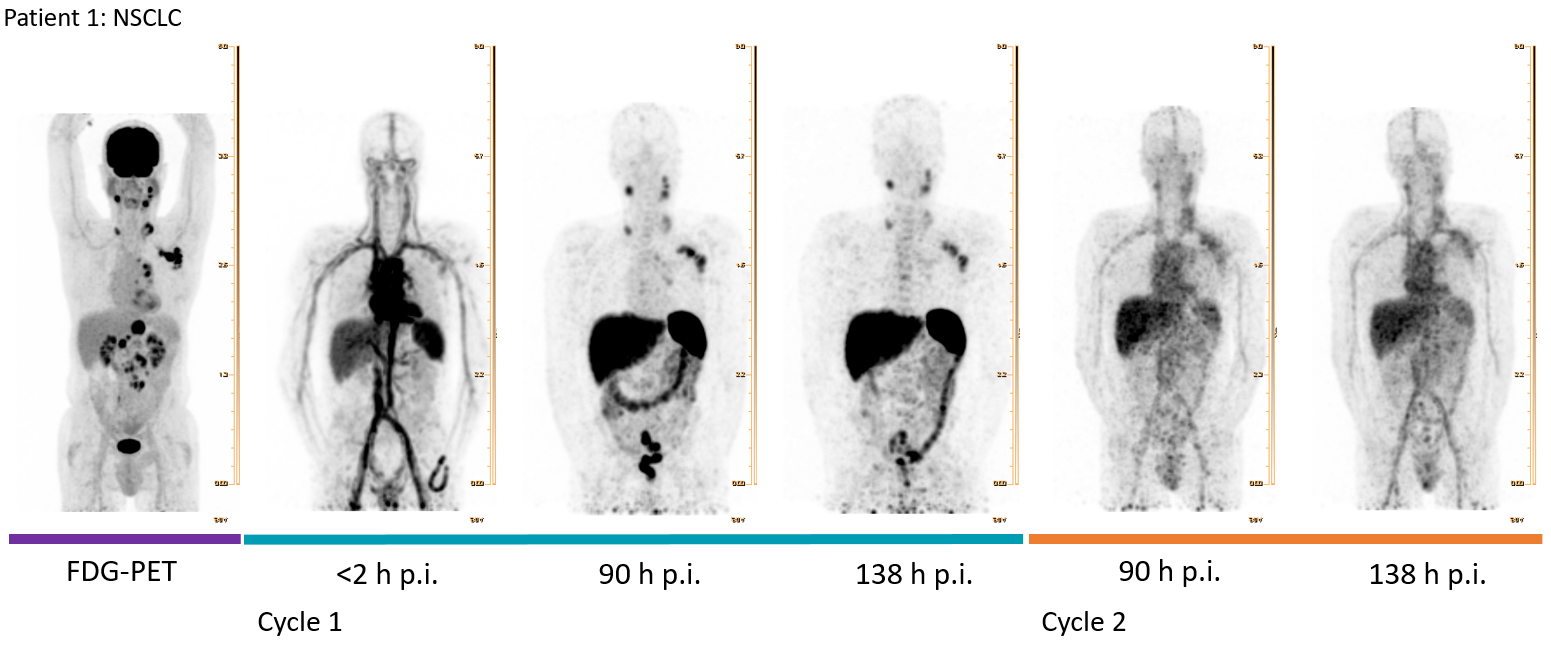


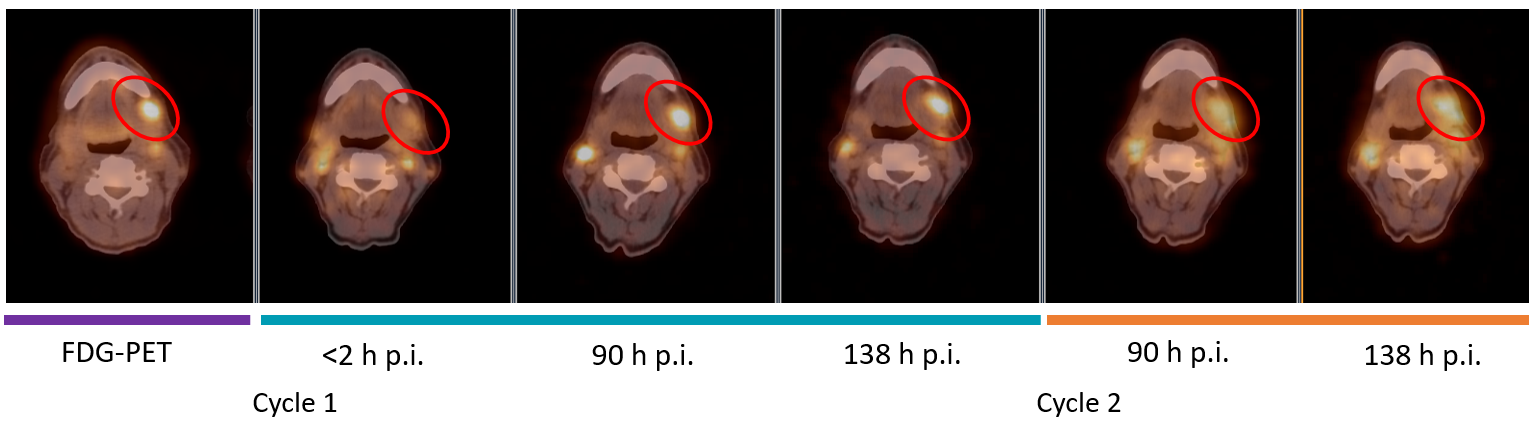


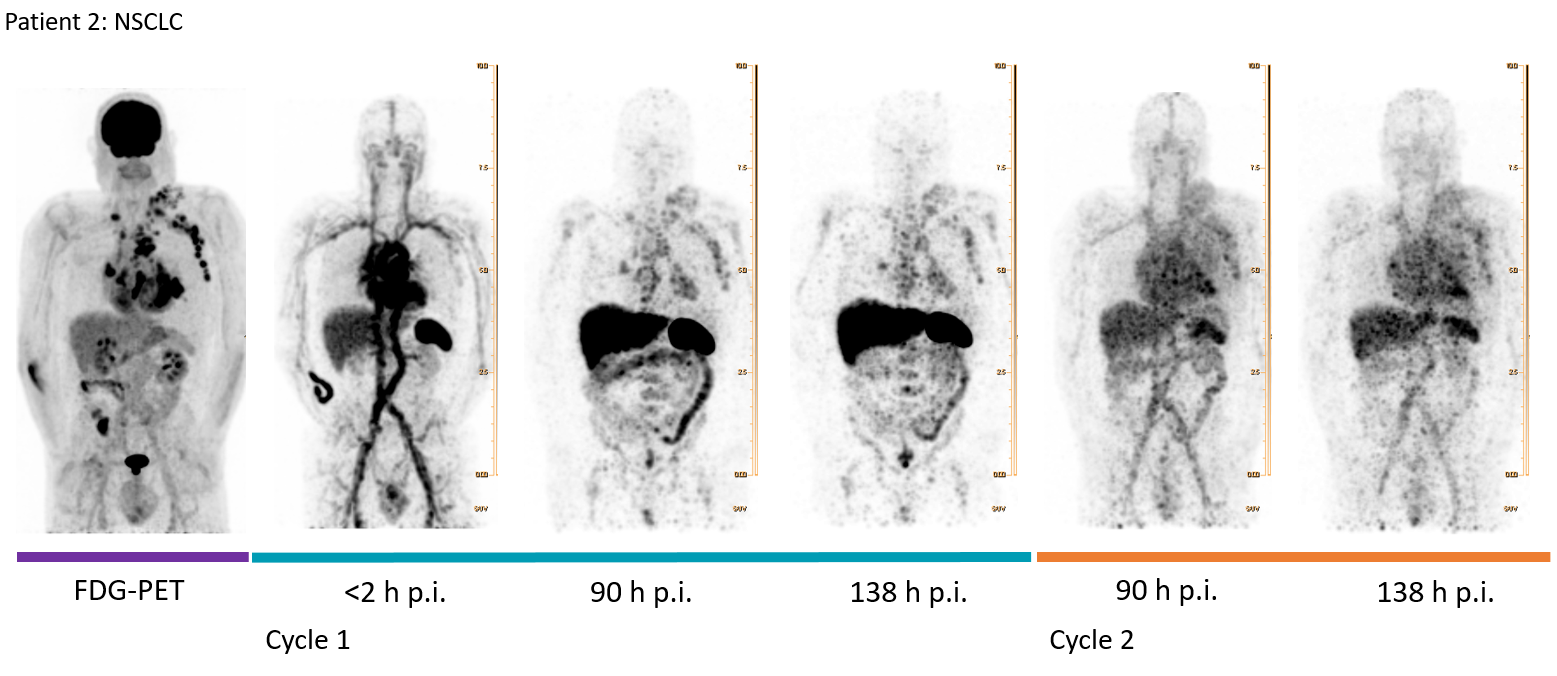

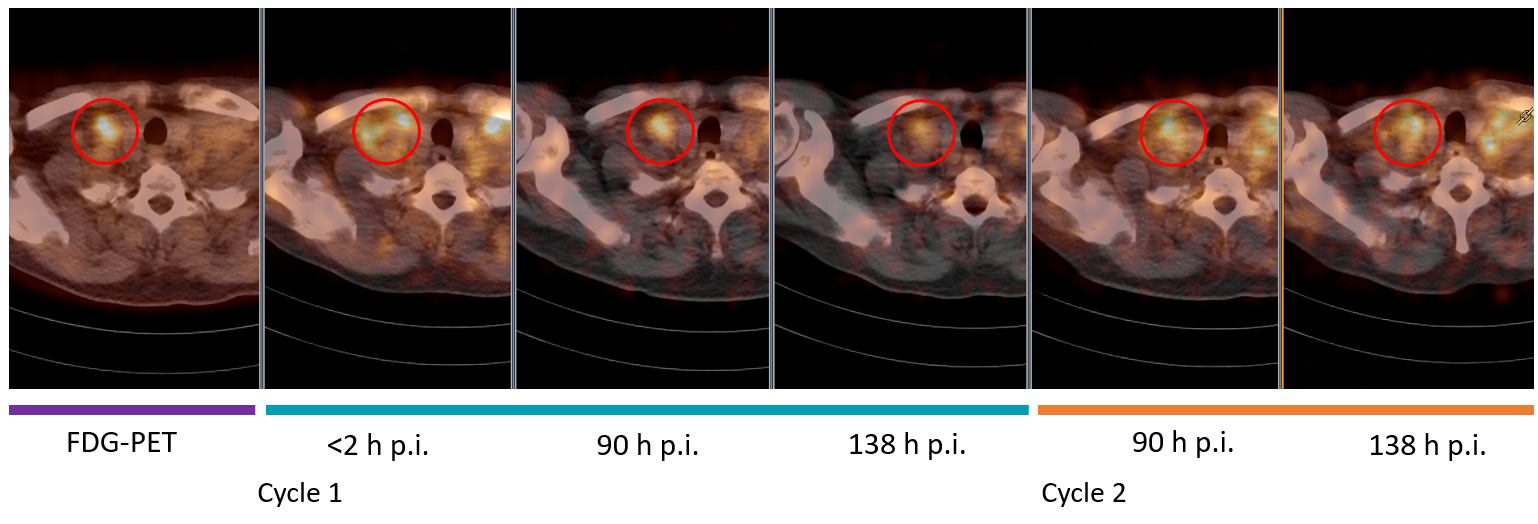


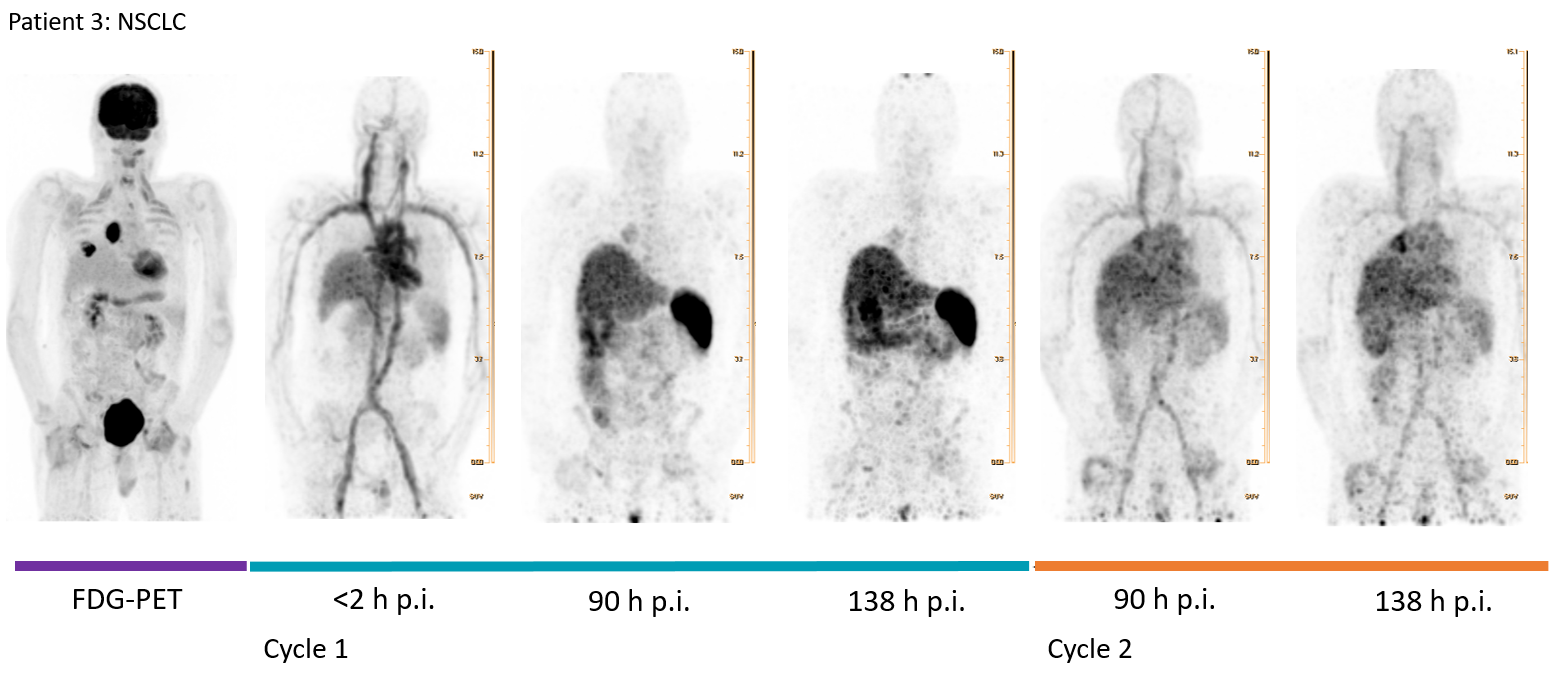


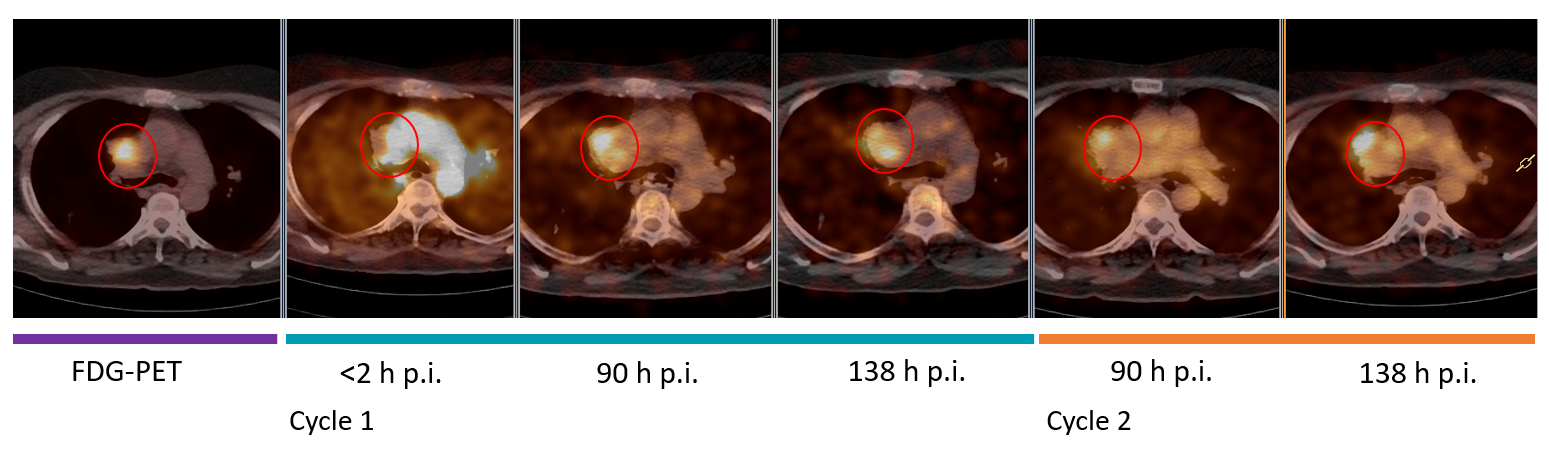

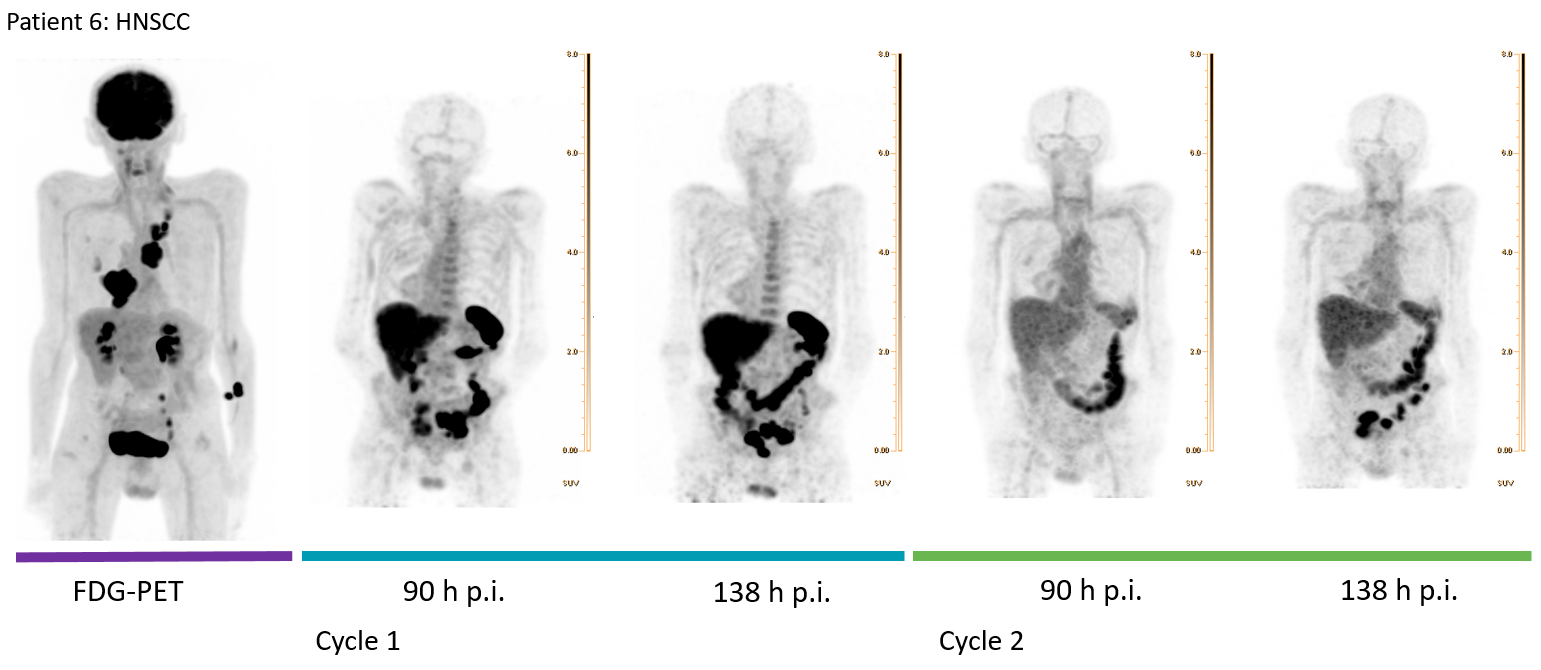

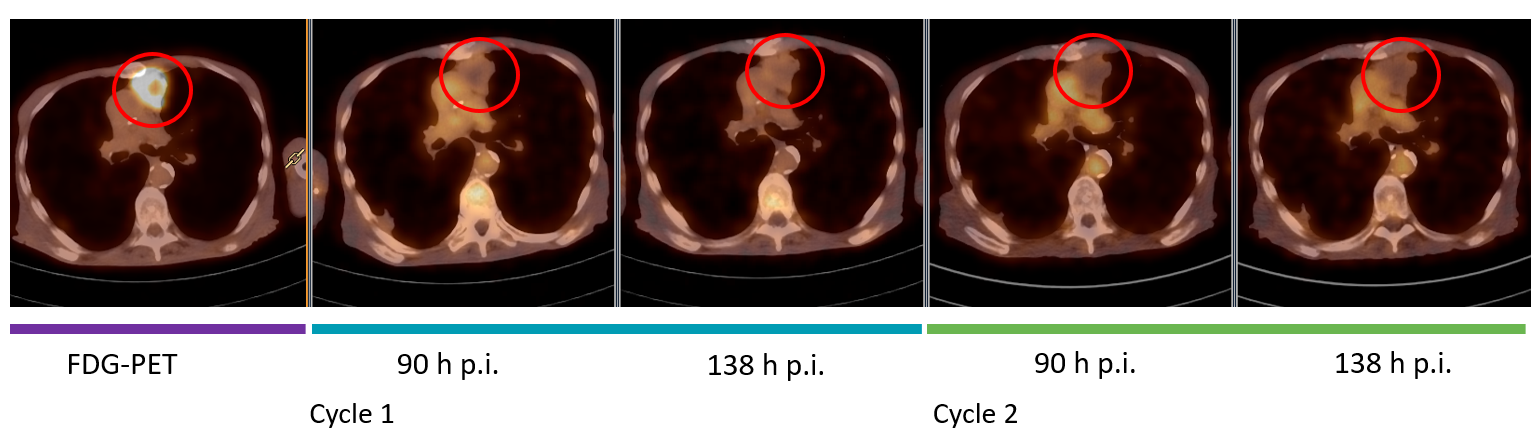


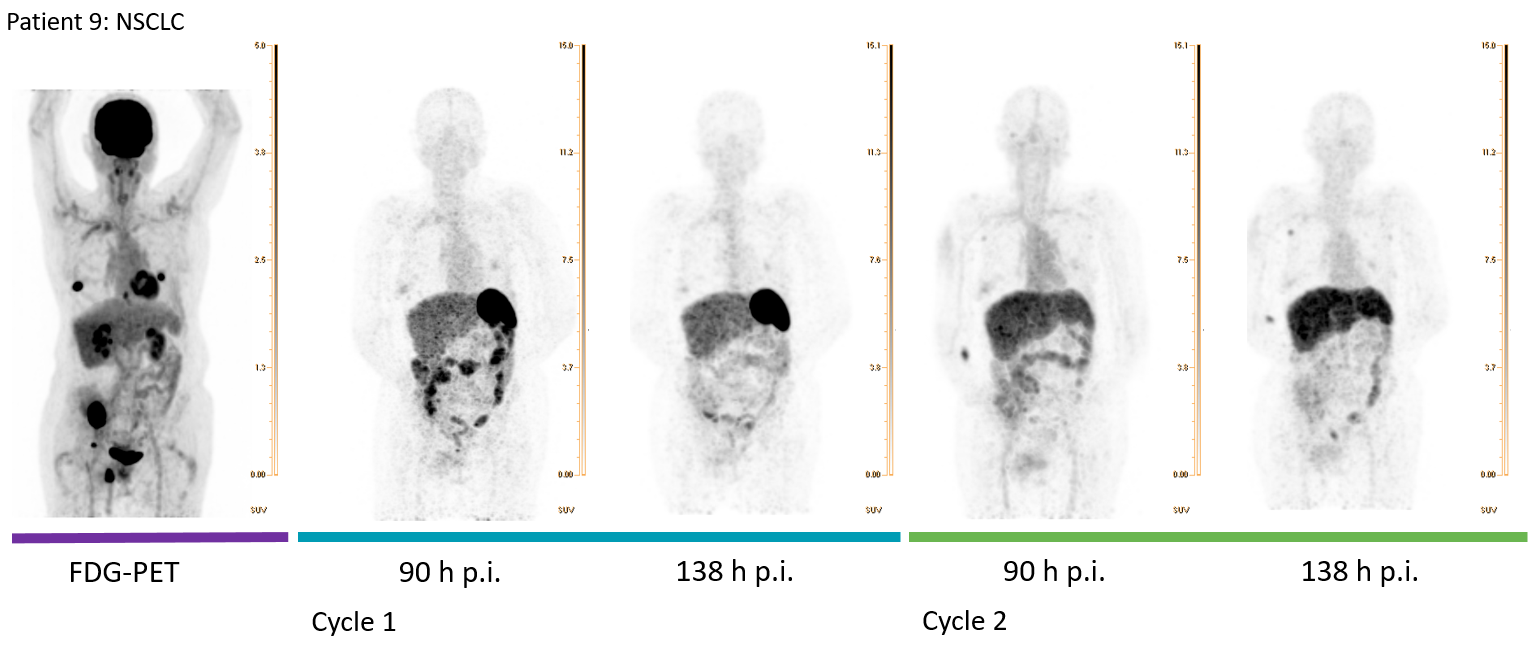

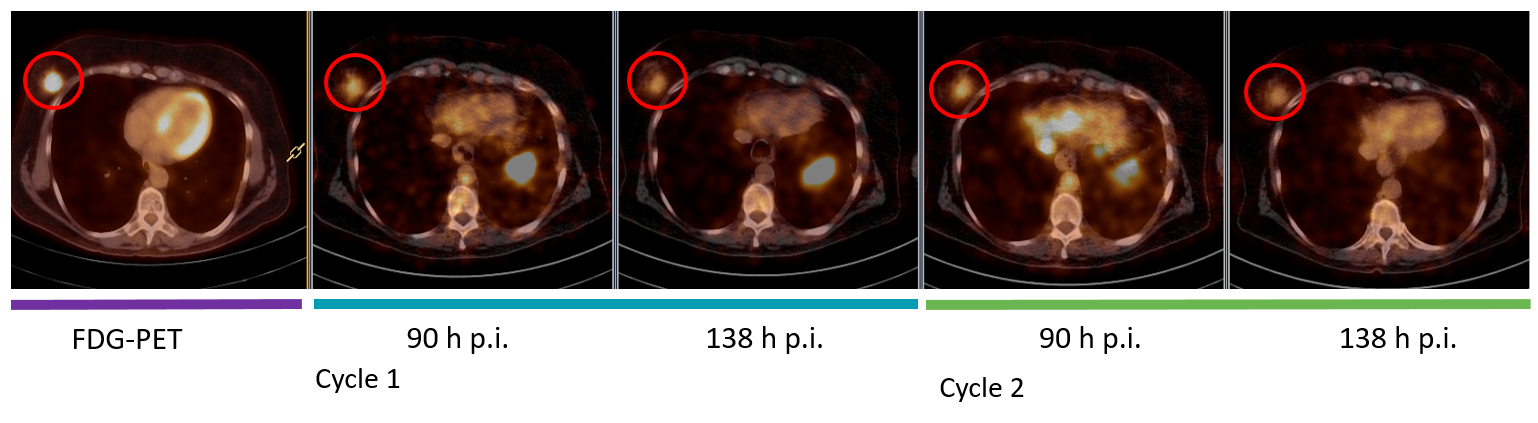

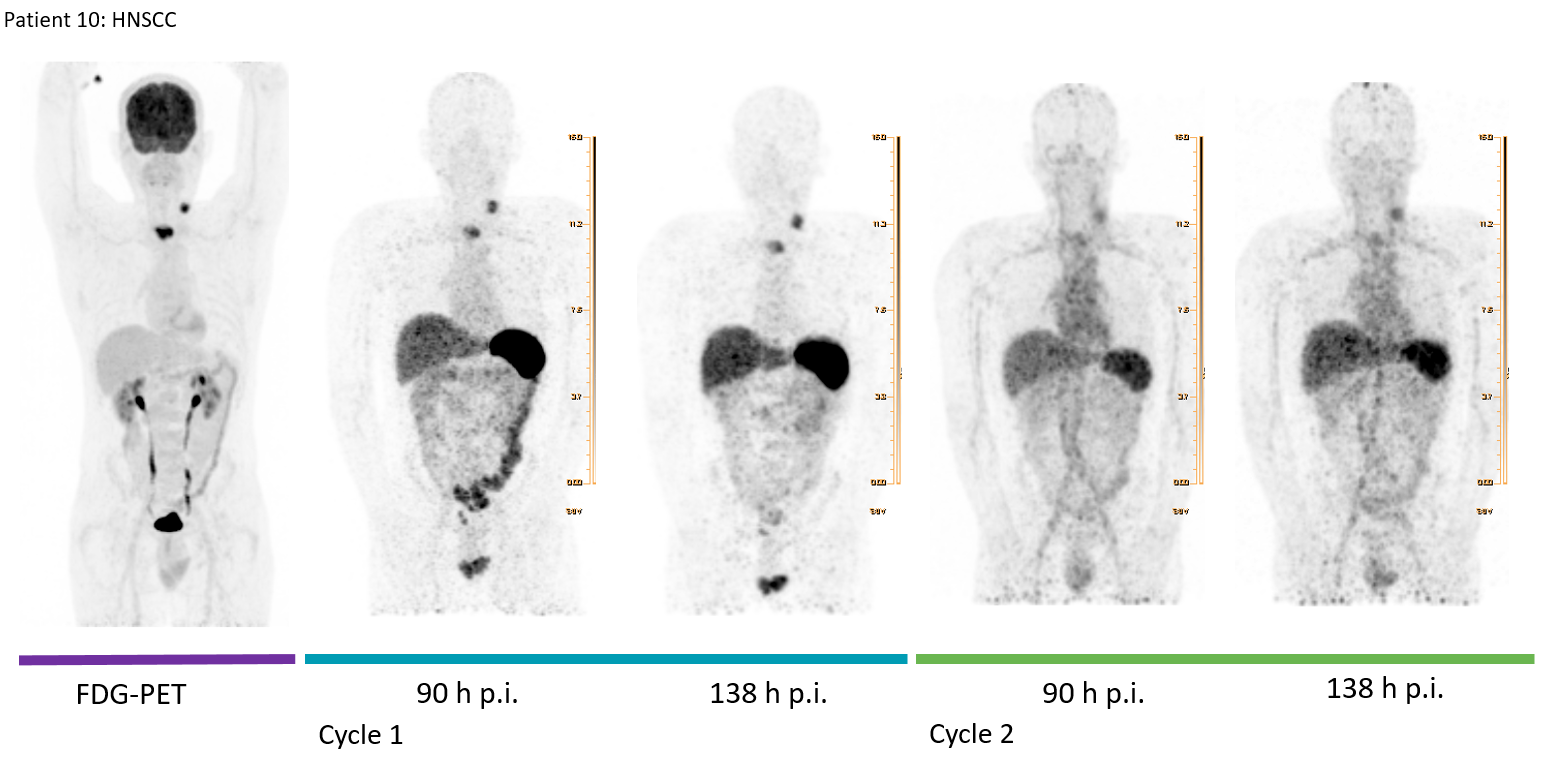

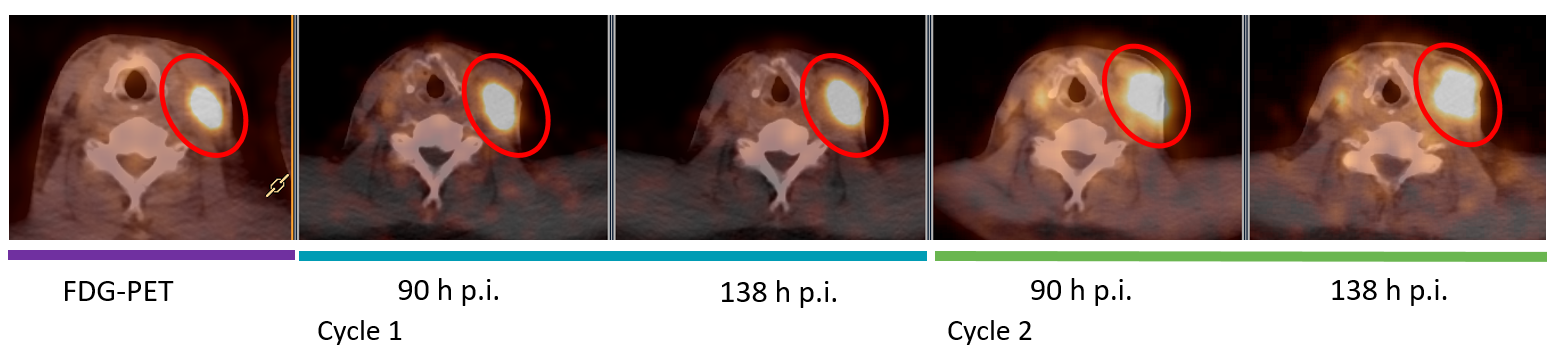
**Supplementary Fig. 4** MIP images of each scan and detailed axial PET-CT fusion images of one tumor lesion per patient. A red circle indicates a tumor lesion.


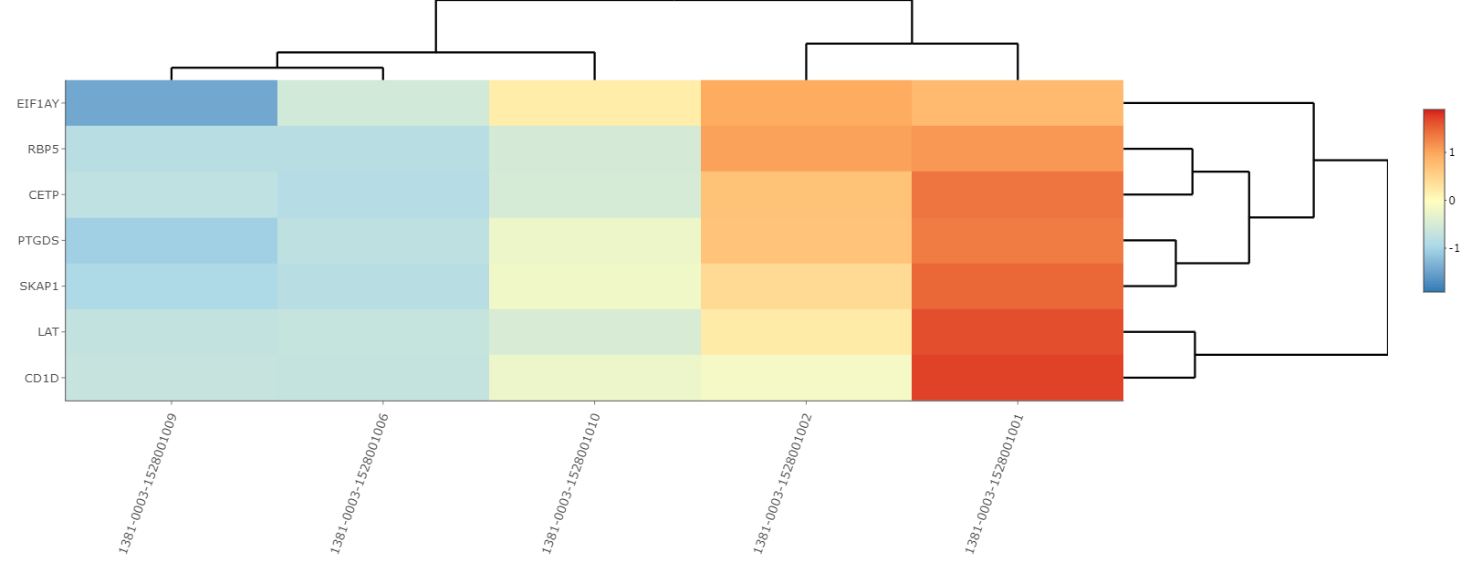


**Supplementary Fig. 5** TLS signature. Heatmap for the TLS signature computed using the GSVA algorithm and based on the signature described in Cabrita et al. (2)*.* *GSVA* gene set variation analysis, *TLS* tertiary lymphoid structure


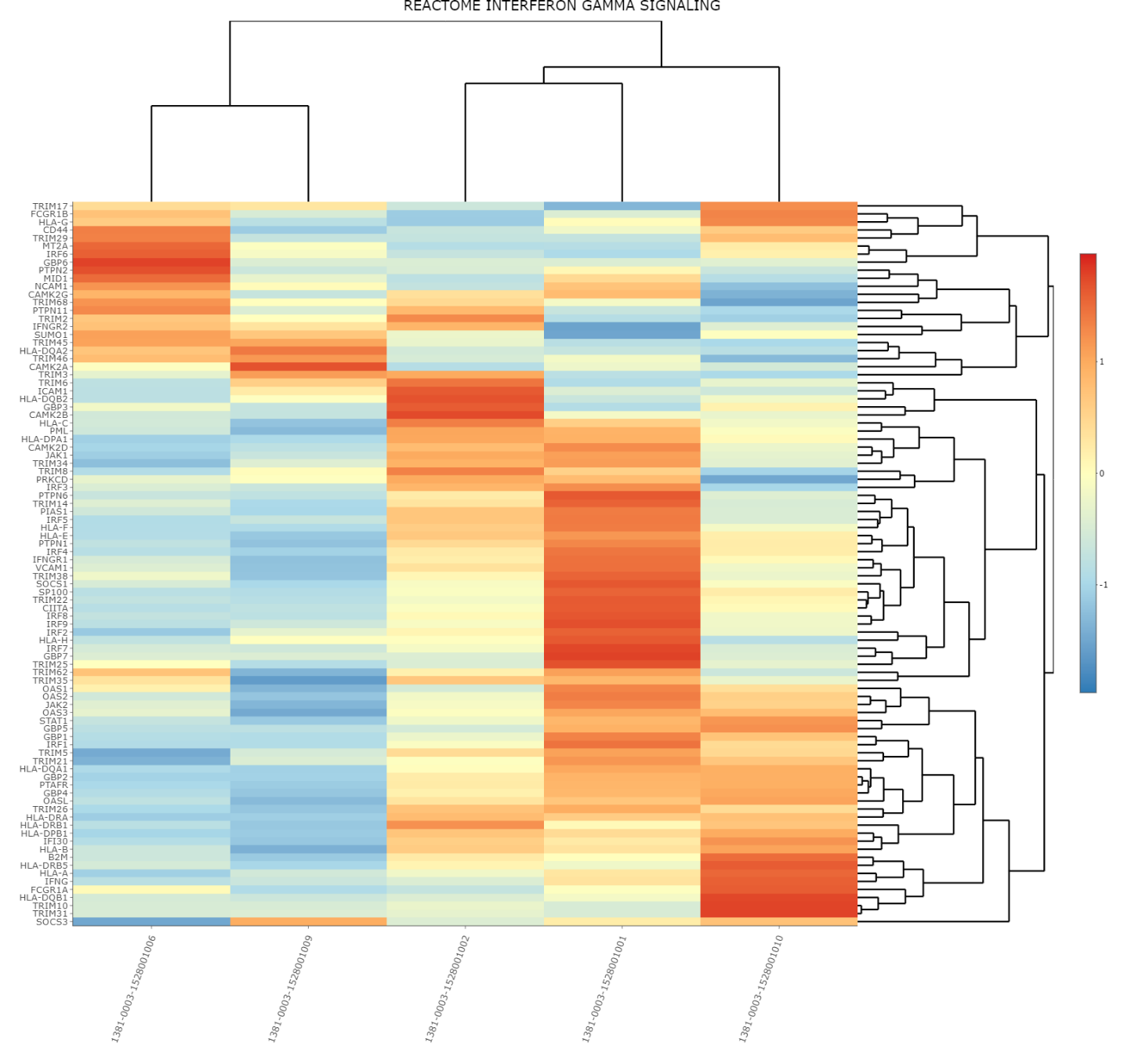

**Supplementary Fig. 6** IFN-gamma signature. Heatmap for IFN-gamma signature values for all 5 patients computed using the GSVA algorithm using the Hallmark IFN-Gamma Response Signature (3). *GSVA* gene set variation analysis, *IFN* interferon

**References**

1. Jauw YWS, O'Donoghue JA, Zijlstra JM, Hoekstra OS, Menke-van der Houven van Oordt CW, Morschhauser F, et al. (89)Zr-immuno-PET: toward a noninvasive clinical tool to measure target engagement of therapeutic antibodies in vivo. J Nucl Med 2019;60:1825-32.

2. Cabrita R, Lauss M, Sanna A, Donia M, Skaarup Larsen M, Mitra S, et al. Tertiary lymphoid structures improve immunotherapy and survival in melanoma. Nature 2020;577:561-5.

3. Liberzon A, Birger C, Thorvaldsdottir H, Ghandi M, Mesirov JP, Tamayo P. The Molecular Signatures Database (MSigDB) hallmark gene set collection. Cell Syst 2015;1:417-25.
